# A transformer-based survival model for prediction of all-cause mortality in patients with heart failure: a multi-cohort study

**DOI:** 10.1038/s41746-025-02296-5

**Published:** 2026-01-08

**Authors:** Shishir Rao, Nouman Ahmed, Gholamreza Salimi-Khorshidi, Christopher Yau, Huimin Su, Nathalie Conrad, Folkert W. Asselbergs, Mark Woodward, Rod Jackson, John GF Cleland, Kazem Rahimi

**Affiliations:** 1 Nuffield Department of Women’s & Reproductive Health, University of Oxford, Oxford, UK; 2 Health Data Research UK, London, UK; 3 Department of Cardiovascular Sciences, Katholieke Universiteit Leuven, Leuven, Belgium; 4 Amsterdam University Medical Centers, Department of Cardiology, University of Amsterdam, Amsterdam, The Netherlands; 5 Institute of Health Informatics, University College London, London, UK; 6 The George Institute for Global Health, University of New South Wales, Newtown, NSW Australia; 7 The George Institute for Global Health, Imperial College London, London, UK; 8 School of Population Health, Faculty of Medical and Health Sciences, University of Auckland, Auckland, New Zealand; 9 British Heart Foundation Centre of Research Excellence, School of Cardiovascular and Metabolic Health, University of Glasgow, Glasgow, UK

**Keywords:** Cancer, Computational biology and bioinformatics, Health care, Medical research, Risk factors

## Abstract

Heart failure (HF) patients have complex health profiles that existing risk models fail to capture. We developed TRisk, a Transformer-based artificial intelligence survival model for predicting mortality using routine electronic health records (EHR) in HF patients. Using UK data from 403,534 HF patients across 1418 English general practices, we trained and validated TRisk and compared it against MAGGIC-EHR, the MAGGIC model adapted for use on routine EHR by substituting variables (e.g. left-ventricular ejection fraction) that are not routinely available. External validation was conducted on 21,767 patients from USA hospitals. In the UK cohort, TRisk achieved a concordance index (*C*-index): 0.845 (95% CI: 0.841, 0.849), outperforming MAGGIC-EHR (*C*-index: 0.728 [0.723, 0.733]) for 36-month mortality prediction. In subgroup analyses, TRisk demonstrated less variability in predictive performance by sex, age, and baseline characteristics compared to MAGGIC-EHR, suggesting less biased modelling. Evaluating TRisk in USA data via transfer learning yielded a *C*-index of 0.802 (0.789, 0.816). Explainability analysis revealed TRisk captured established risk factors while identifying underappreciated ones, particularly cancers and hepatic failure, with cancers maintaining prognostic utility even a decade before baseline. TRisk provides more accurate, well-calibrated mortality prediction using routine data across international healthcare settings, demonstrating potential for improved risk stratification in patients with HF.

## Introduction

Heart failure (HF) is a complex clinical syndrome with a highly variable prognosis^1^. Risk prediction of outcomes over a few days or over many decades is relatively accurate in HF, but predicting medium-term outcomes over years is often difficult. Prediction in this time range is, however, important for initiating interventions, effective communication and auditing the quality of care across organisations^2,3^.

While risk assessment approaches ranging from simpler single markers (e.g. left ventricular ejection fraction [LVEF]) to more comprehensive models like the Meta-Analysis Global Group in Chronic (MAGGIC) HF have been useful, limitations remain^3^. Many rely on data from specialised tests that are resource-intensive to obtain and are typically gathered after clinical suspicion of HF deterioration has been raised. Moreover, these models offer relatively modest discrimination (i.e. <0.8 concordance index) for select outcomes with poor positive predictive value (PPV) and sensitivity^3–9^. While they focus primarily on including predictors of cardiovascular function, current models fail to capture the complex, evolving nature of multi-factorial risk, overlooking important comorbidities and interventions that influence outcomes. In fact, complex multimorbidity, rather than HF itself, contributes to over 40% of mortality in HF patients, highlighting a significant gap in current risk assessment approaches^10–12^. Given these limitations, current models have consequently seen limited clinical adoption, with HF guidelines emphasising the need for more robust models with transparent reporting of performance metrics^6,7,13^.

The widespread adoption of electronic health records (EHR) offers the potential for developing sophisticated risk assessment models that capture dynamic, multi-factorial patient risk profiles. The Bidirectional EHR Transformer (BEHRT) and its survival modelling variant, the Transformer-based Risk assessment survival model (TRisk) represent a breakthrough in efficient patient health modelling using large-scale EHR^14,15^. These artificial intelligence (AI) models capture the complete patient journey by processing various medical data types—diagnoses, medications, procedures, and test results—advancing prognostication research across multiple fields, including cardiology, oncology, and pulmonology^14–17^. Furthermore, TRisk is designed to operate on internationally standardised data (e.g. SNOMED), demonstrating promise for application on global data. Motivated by this, we developed and validated TRisk for the prediction of all-cause mortality in HF patients using the United Kingdom (UK) and the United States of America (USA) EHR datasets.

## Results

### Overview

Using a UK cohort of patients with HF, we derived TRisk and a version of the MAGGIC Cox proportional hazards model adapted for use on primary and secondary care EHR (termed MAGGIC-EHR) as certain variables (e.g. LVEF) are not readily accessible for modelling in this setting^3,15^. All models were validated in a separate cohort of UK patients with HF. To assess model generalisability and model utility in a dataset from another geographical setting, external validation of TRisk for all-cause mortality prediction was conducted in a cohort of patients with HF from the USA. To understand the decision-making processes of the TRisk AI model, we analysed the input EHR encounters that the model found important for conducting prediction across both cohorts.

### UK data cohort characteristics and model development

For the UK data, we used the Clinical Practice Research Datalink (CPRD) Aurum dataset, which contains routine electronic health records from English general practices covering approximately 20% of the UK population^18^. Patients with a HF diagnosis recorded in primary or secondary care between 40 and 90 years of age between January 1 2005 and December 31 2019 from 1,418 GP practices across England were included for analyses; for all model development, 1063 (75%) practices were randomly assigned to the derivation dataset for model training and 355 (25%) to an external validation dataset.

In this dataset, we analysed 403,534 HF patients (99,382 in validation) with a median follow-up of 9 months (interquartile interval [IQI]: 2-29) (Fig. S1). Median age was 79 years (IQI: 70–85), with approximately one-third having diabetes, myocardial infarction history, or beta-blocker prescriptions at baseline (Table 1). Approximately 44% of patients died within four years.

**Table 1:** Population characteristics for derivation and validation datasets in the UK data study at baseline

	Derivation (304,152 patients)	Validation (99,382 patients)
Women (%)	139,762 (46.0)	45,444 (45.7)
Median age (years) (IQI)	79 (70, 85)	79 (70, 85)
Smoking status^†^		
No (%)	133,284 (43.8)	43,605 (43.9)
Ex (%)	131,651 (43.3)	42,927 (43.2)
Yes (%)	39,217 (12.9)	12,850 (12.9)
Median BMI (kg/m^2^) (IQI)^†^	27.5 (24.0, 31.8)	27.5 (24.0, 31.7)
Median SBP (mmHg) (IQI)^†^	134.0 (124.9, 143.1)	133.9 (124.9, 143.1)
Median sodium (mmol/L) (IQI)^†^	139.6 (137.5, 141.3)	139.5 (137.5, 141.2)
Median creatinine (µmol/L) (IQI)^†^	91.3 (76.0, 113.4)	91.8 (76.0, 113.8)
HF subtype (by LVEF status)		
Unknown (%)	225,948 (74.3)	73,564 (74.1)
HFpEF (%)	12,997 (4.3)	4350 (4.4)
HFrEF (%)	65,207 (21.4)	21,468 (21.6)
<18 months since incident HF (%)	208,977 (68.7)	68,279 (68.7)
NYHA classification^†^		
I (%)	76,044 (25.0)	24,700 (24.9)
II (%)	143,318 (47.1)	46,661 (47.0)
III (%)	76,591 (25.2)	25,264 (25.4)
IV (%)	8199 (2.7)	2757 (2.8)
Disease history		
Diabetes (%)	91,740 (30.2)	29,659 (29.8)
COPD (%)	81,100 (26.7)	27,099 (27.3)
AF (%)	146,493 (48.2)	48,033 (48.3)
Stroke (%)	70,675 (23.2)	22,604 (22.7)
MI (%)	86,429 (28.4)	28,500 (28.7)
Prescription history		
Beta-blockers (%)	102,741 (33.8)	34,258 (34.5)
ACE-Is/ARBs (%)	19,5311 (64.2)	63,718 (64.1)
Procedure history		
PCI (%)	33,726 (11.1)	11,059 (11.1)
CABG (%)	18,190 (6.0)	6078 (6.1)

*%* percent, *IQI* interquartile interval (25th and 75th percentiles), *BMI* body mass index, *SBP* systolic blood pressure, *HFpEF* heart failure-preserved ejection fraction, *HFrEF* heart failure-reduced ejection fraction, *LVEF* left-ventricular ejection fraction, *NYHA* New York Heart Association, *COPD* Chronic obstructive pulmonary disease, *AF* atrial fibrillation, *MI* myocardial infarction, *ACE-I* angiotensin-converting-enzyme inhibitors, *ARB* angiotensin receptor blockers, *PCI* percutaneous coronary intervention, *CABG* coronary artery bypass graft.

^†^Indicates missing variables; BMI (26.7% missingness), smoking status (14.0%), SBP (4.0%), creatinine (9.1%), NYHA (96.6%), and sodium (9.7%).

The TRisk model for prognostication of 36-month mortality in patients with HF was derived on the derivation dataset. The TRisk model processes patients’ complete medical history up to baseline as timestamped sequences, incorporating 2639 distinct diagnosis codes, 633 medication codes, and 852 procedure codes from linked primary and secondary care records. The model considers relative ordering and patient age at each encounter, providing rich longitudinal annotations without imputation of missing values (Fig. S2; model hyperparameters in Table S1)^15^. For benchmark modelling, the MAGGIC-EHR model was adapted for use with primary and secondary care EHR data^3^.

**Fig. 1:**
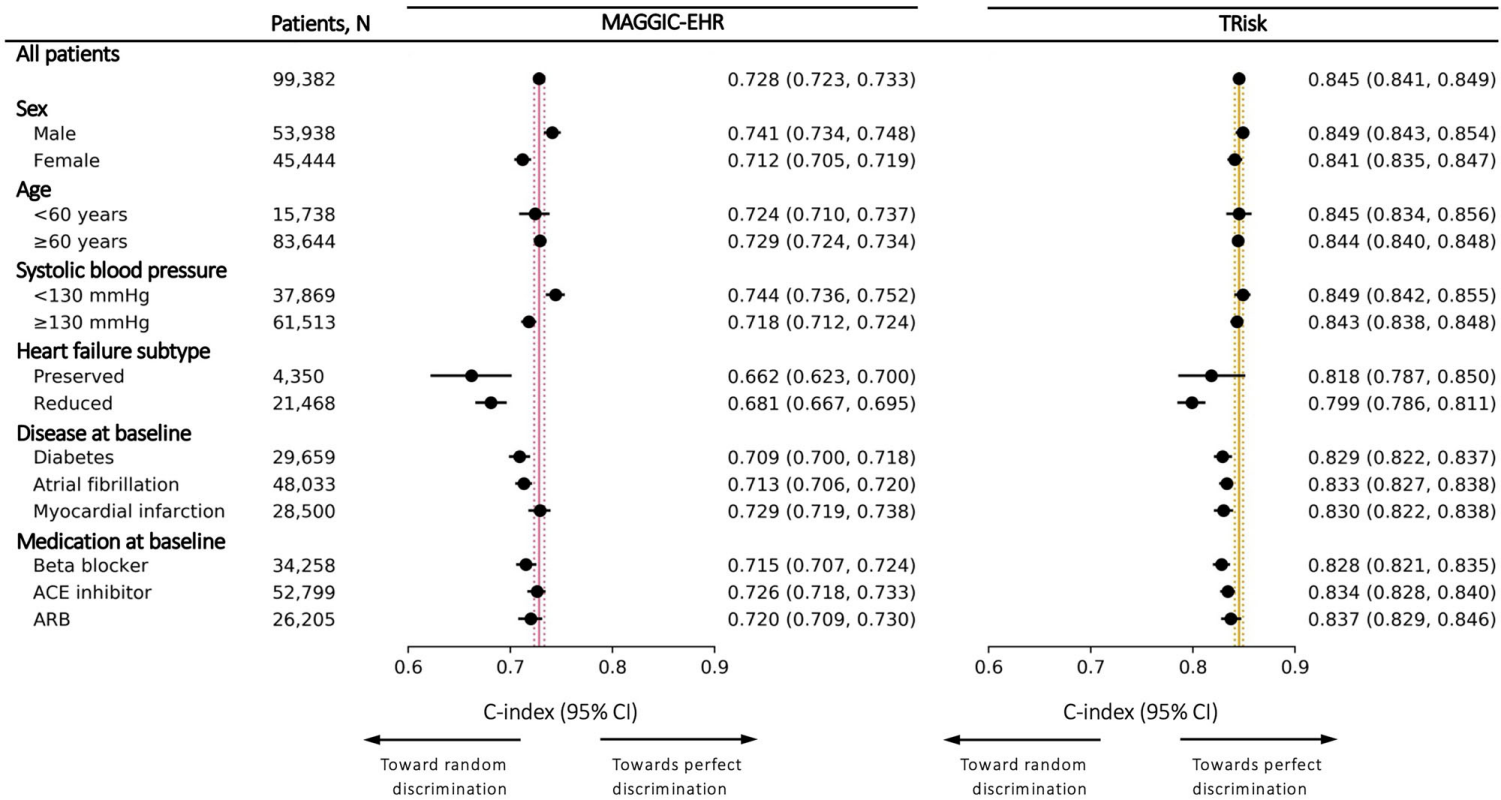
Models’ discrimination by concordance index (*C*-index) and associated 95% confidence intervals (CI) in overall cohort and subgroups for 36-month all-cause mortality risk prediction on UK validation data. Maroon and gold lines represent the *C*-index on “all patients” in the validation cohort for MAGGIC-EHR and TRisk, respectively. These solid and dotted coloured lines represent the mean and 95% CI boundaries of the overall cohort *C*-index for each model. These lines are provided to visually demonstrate deviation in subgroup discrimination performance from overall cohort performance for each model. ACE angiotensin-converting enzyme, ARB angiotensin receptor blocker.

**Fig. 2:**
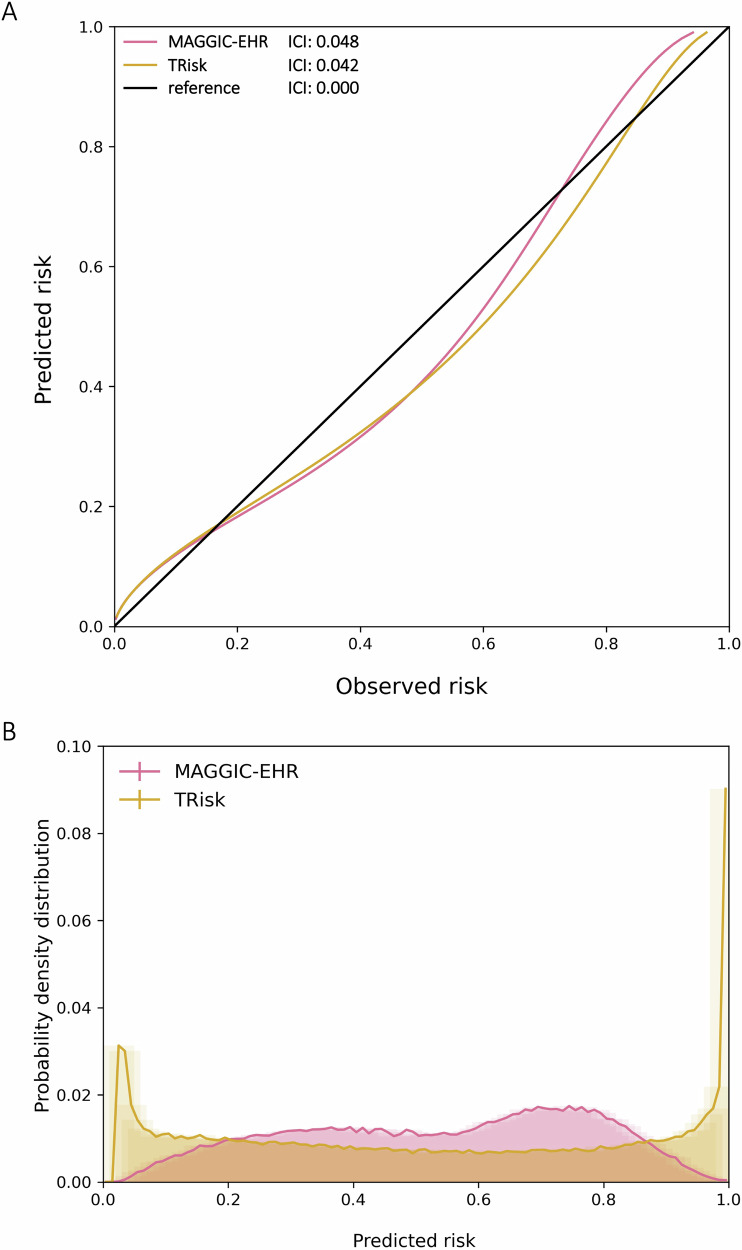
Calibration curves and integrated calibration indices (ICI) and distribution of predicted risk of models for 36-month all-cause mortality risk prediction on UK validation data. Calibration curves with ICI (**A**) and distribution of predicted risk (**B**) are presented for all models. For (**A**) ICI, lower is better, with reference (black line) presenting the optimal ICI of 0.0.

In terms of discrimination, TRisk achieved a higher Concordance-index (*C*-index: 0.845; 95% CI [0.841, 0.849]) than MAGGIC-EHR (0.728; 95% CI [0.723, 0.733]), along with a higher area under the precision-recall curve (AUPRC; Figs. 1 and S3 and Table S2). The subgroup discrimination analyses yielded that the TRisk outperformed MAGGIC-EHR across key subgroups (Fig. 1 and Table S3) and importantly presented lesser deviation from the overall cohort discrimination across subgroup analyses as compared to MAGGIC-EHR. Both models were well calibrated as evidenced by the general affinity of calibration curves to the reference curve (Figs. 2 and S4) and low integrated calibration index (ICI) estimates (Table S4). In terms of predictive distribution, TRisk demonstrated two peaks in the lowest and highest ends of the predicted risk spectrum implying more nuanced stratification of risk as compared to the MAGGIC-EHR model, which showed a unimodal distribution with a high concentration of predictions towards the middle (Figs. 2 and S4). Decision curve analysis showed that TRisk provided significantly greater net benefit than other strategies (Fig. S4C) across the spectrum of clinically relevant thresholds (up to ~0.6).

Investigating the performances of additional models, TRisk outperformed MAGGIC-EHR+ (i.e. MAGGIC-EHR with additional predictors for fairer comparison with TRisk), which performed similarly to MAGGIC-EHR (Figs. S3 and S4; and Tables S2–S4). Additionally, ablation analyses were performed on the TRisk model to assess the contribution of individual components to its discrimination and calibration performance. The analyses revealed that TRisk showed improved performance in terms of discrimination and calibration as compared to variations of TRisk without specific modelling components (Supplementary Results: Ablation analyses; Table S5). Furthermore, random survival forest modelling with TRisk vocabulary demonstrated modest improvement in discrimination upon the benchmark statistical models (*C*-index: 0.754 [0.749; 0.759]) with acceptable calibration (Tables S6 and S7). In additional analyses of 12-month all-cause mortality prediction, TRisk similarly outperformed benchmark statistical models (Fig. S5 and Table S8).

**Fig. 3:**
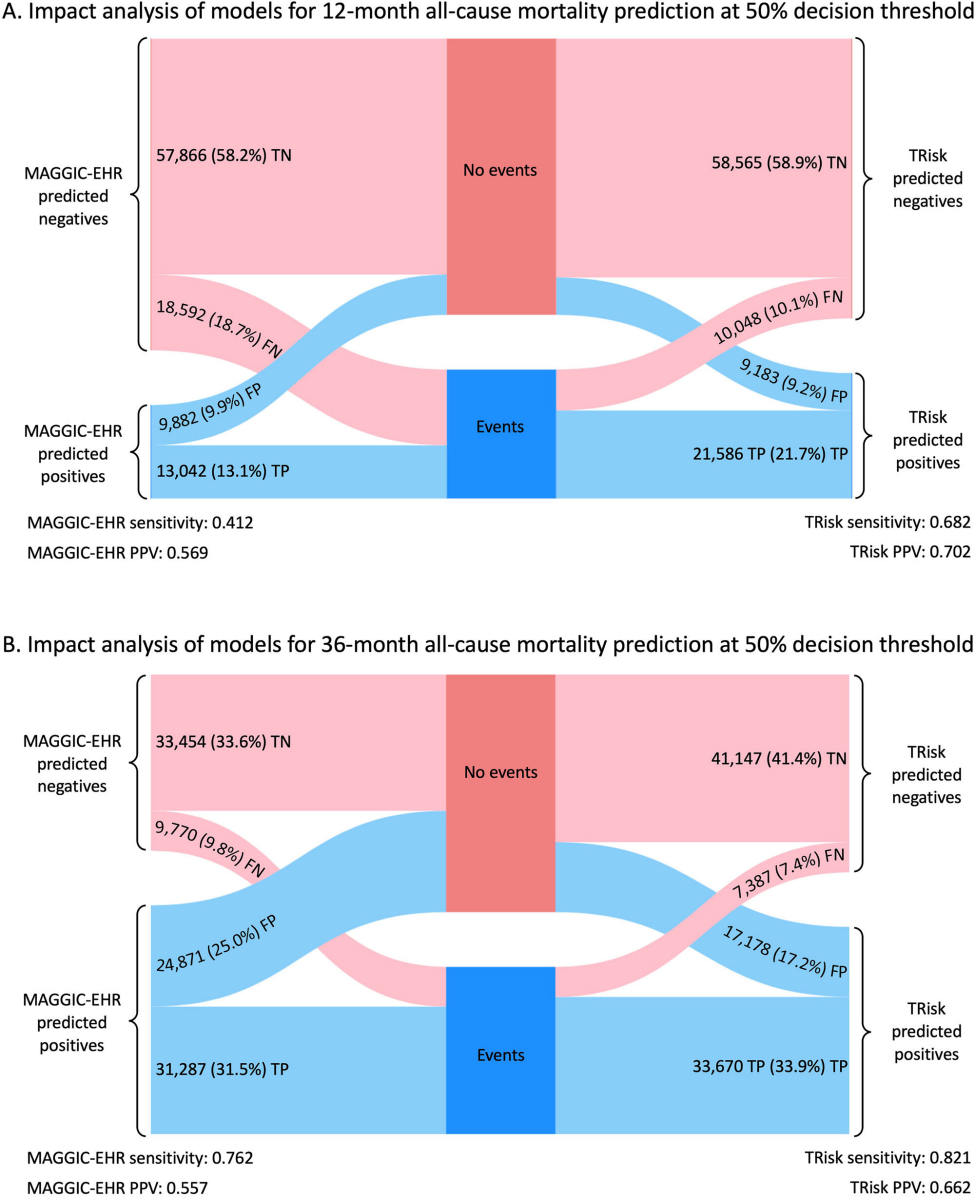
Impact analyses at the 50% decision threshold for 12- and 36-month all-cause mortality prediction on UK validation data. Impact analyses, comparing TRisk and MAGGIC-EHR, at the 50% decision threshold for (**A**) 12- and (**B**) 36-month all-cause mortality prediction on the UK validation data. Sankey diagrams compare predicted outcomes between the models, showing how patient classification compares to actual outcomes, “Events” (i.e. patients who suffered mortality) and “No events “ (i.e. patients who did not suffer mortality) categories at a 50% threshold (denoted as dark blue and red, respectively). Light blue and red colours represent positive and negative predictions, respectively. TP true positive, TN true negative, FP false positive, FN false negatives.

Furthermore, in line with research recommendations by HF clinical guidelines^13^, impact analyses were undertaken at various thresholds to assess model performance at various decision thresholds. At the 50% decision threshold, TRisk reduced false positives and false negatives by 9% (699 patients) and 46% (8544), respectively, for 12-month prediction, and by 31% (7693) and 24% (2383), respectively, for 36-month prediction, as compared to MAGGIC-EHR (Fig. 3 and Table S9). Fewer false positives and false negatives directly translated into TRisk outperforming MAGGIC-EHR in both positive predictive value (PPV) and sensitivity, showing increases of 0.133 and 0.270 for 12-month prediction, and 0.105 and 0.059 for 36-month prediction, respectively. In extended impact analyses, while TRisk outperformed MAGGIC-EHR in terms of PPV and sensitivity at 50% and 75% threshold, it secured higher PPV but lower sensitivity at the 25% threshold as compared to benchmark modelling (Table S9; further elaboration in Supplementary Results: Impact analyses on CPRD validation dataset).

### Analyses of USA data

For USA data, we analysed the Medical Information Mart for Intensive Care IV (MIMIC-IV) dataset from Beth Israel Deaconess Medical Center, Massachusetts, USA^19^. On this dataset (Fig. S6), 21,767 patients with HF diagnosed between 2008 and 2019 were selected for analysis, with 60% of the identified cohort allocated to the validation dataset and the remaining 40% in the fine-tuning dataset. The cohort had a median follow-up of 7 (IQI: [2, 13]) months (Table S10). MIMIC-IV cohort had a similar age distribution, smoking patterns, and prevalence of diabetes, chronic obstructive pulmonary disease, and atrial fibrillation as compared to the CPRD cohort (Table S10).

**Fig. 4:**
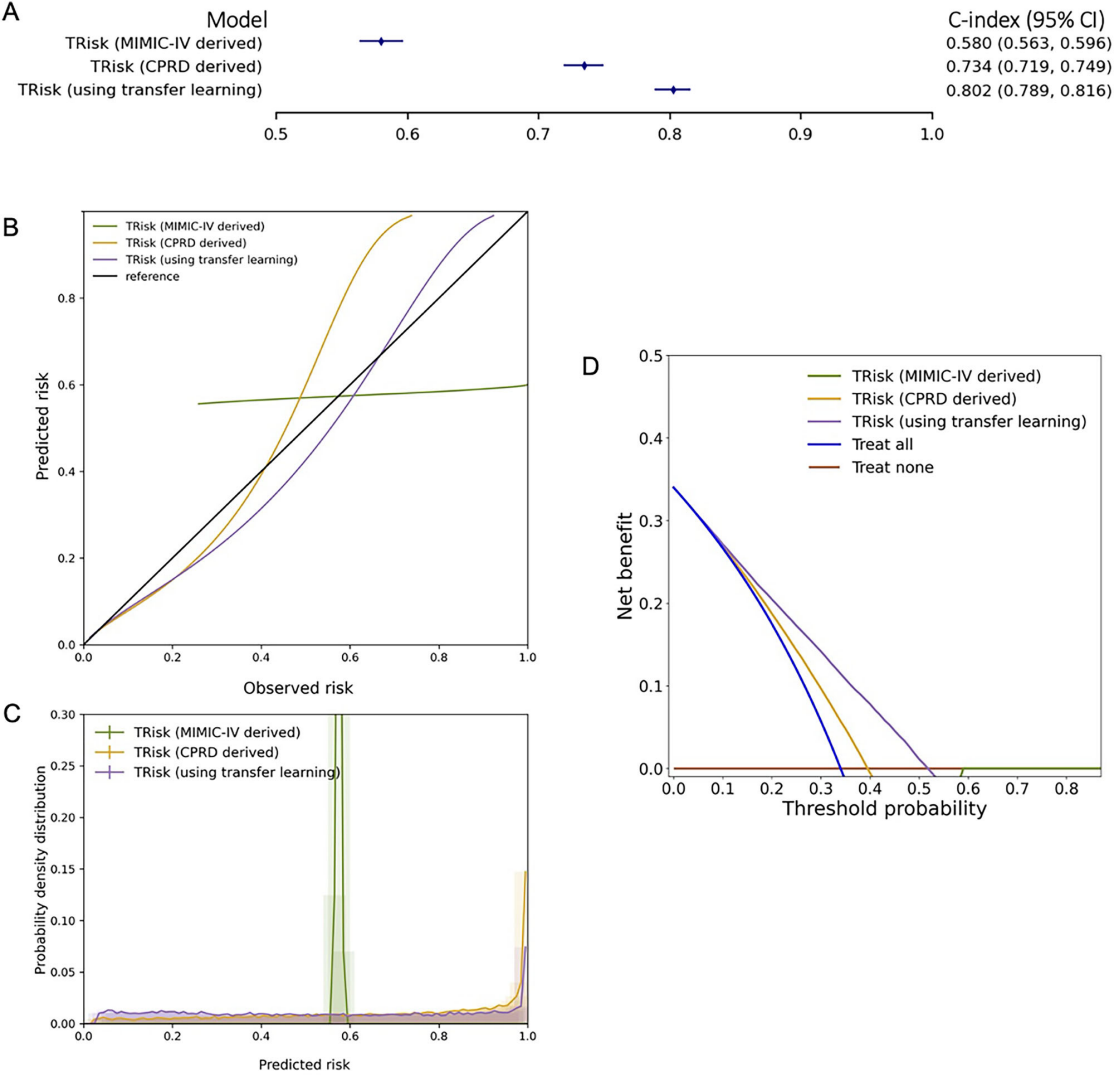
Discrimination performance, calibration, predicted risk distribution, and decision curve analysis of models for 36-month all-cause mortality risk prediction on USA validation data. **A** Discriminative performance of all models, presented as *C*-index with 95% CIs in a forest plot, for 36-month risk prediction of all-cause mortality. **B** Calibration curves, **C** distribution of predicted risk, and **D** decision curve analyses are shown for all models. Decision curve analysis (including censored observations) has been conducted for all models, with threshold probability on the *x*-axis and net benefit on the *y*-axis, representing the difference between the proportion of true positives and false positives weighted by the odds of the respective decision threshold. “TRisk (MIMIC-IV derived)” is randomly initialised and trained on the MIMIC-IV fine-tuning dataset. “TRisk (CPRD derived)” is trained on the CPRD derivation cohort. “TRisk (using transfer learning)” is trained on the CPRD derivation cohort and fine-tuned on the MIMIC-IV fine-tuning dataset. All models are validated on the MIMIC-IV validation dataset.

In analyses of the validation dataset for 36-month mortality prediction, we compared three forms of the TRisk model on the MIMIC-IV validation dataset: TRisk trained on CPRD, TRisk trained from scratch on the fine-tuning dataset, and TRisk initially trained on CPRD then further fine-tuned on the fine-tuning dataset. We found the transfer learning variant of TRisk demonstrated a superior *C*-index (Fig. 4) of 0.802 (0.789, 0.816) compared to other TRisk modelling strategies with similar superiority in AUPRC (Fig. 4A and Table S11). Also, it was found to be better calibrated across the full spectrum of risk (Fig. 4B and Table S12) with appropriate stratification of high-risk individuals as evidenced by a peak in the high end of the risk spectrum (Fig. 4C). Decision curve analyses additionally demonstrated that the transfer learning TRisk variant provided greatest net benefit across all relevant thresholds (Fig. 4D). All findings were preserved in analyses of 12-month mortality prediction (Fig. S7 and Tables S11 and S12; extended results are in Supplementary Results: Analyses on MIMIC-IV validation dataset).

### Explainability analyses

To understand TRisk’s decision-making processes, we conducted explainability analysis using integrated gradients methodology on validation cohorts of both UK and USA datasets^20^. This analysis revealed that TRisk successfully identified validated risk factors as contributing to mortality risk across both cohorts (Fig. S8).

**Fig. 5:**
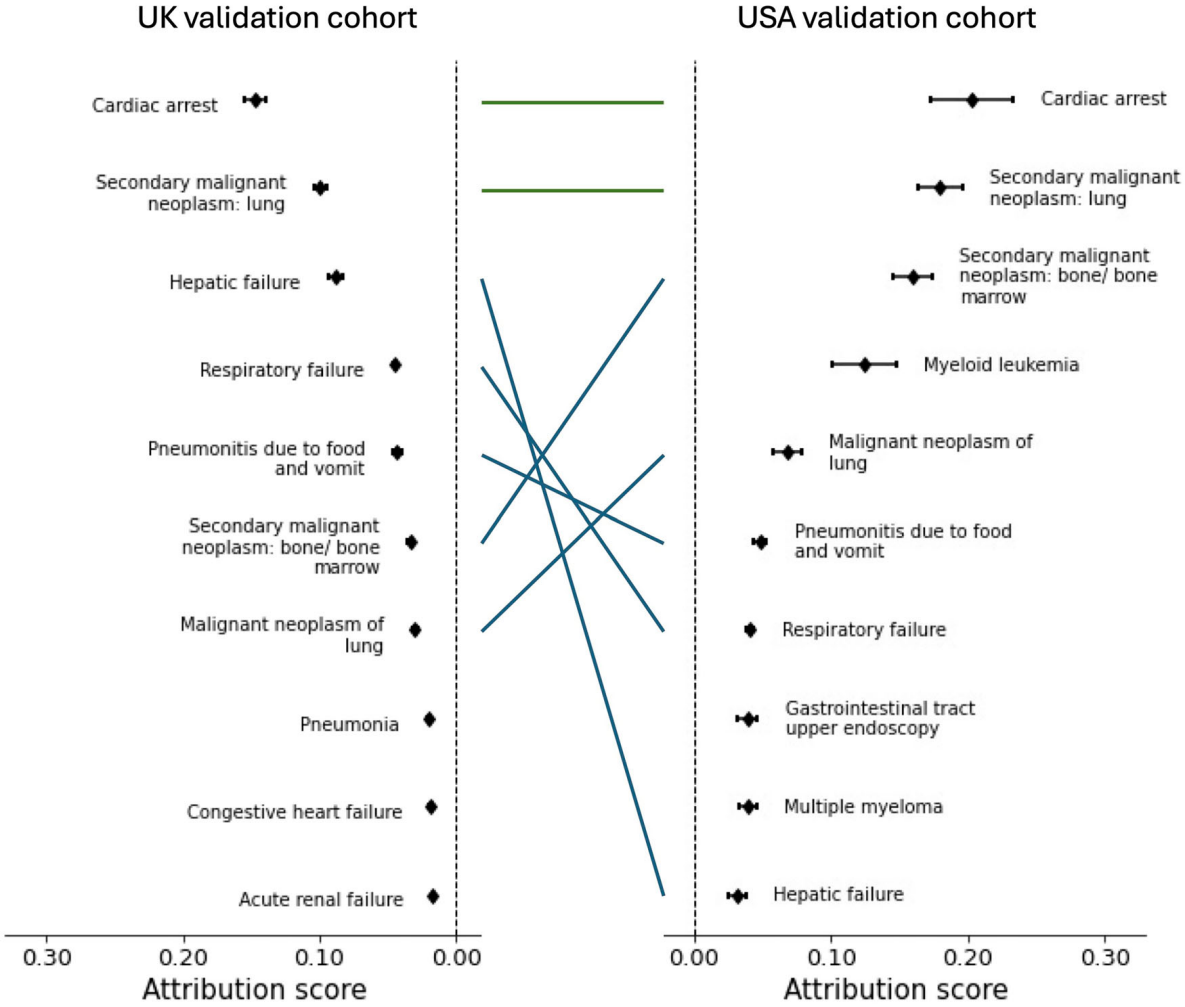
Average contribution for the top ten encounters found as most contributing to mortality risk prediction on the UK and USA validation cohort datasets. Point estimates of contribution values and associated 95% CIs are presented for each encounter. We investigated encounters with top ten contribution scores as captured by our TRisk model across the UK (left) and the USA (right) datasets. The green line denotes that both encounter and rank were preserved between validation analyses; the blue line denotes that only encounter was preserved.

Among the top ten contributing encounters identified by explainability analysis, “Cardiac arrest” and “Secondary malignant neoplasm of lung” were consistently ranked the top two in both the UK and the USA cohorts. Five others appeared among the top-ten contributors in both cohorts, but with varying ranks. These included “hepatic failure”, “respiratory failure”, “pneumonitis due to food and vomit”, “secondary malignant neoplasm of bone/bone marrow”, and “malignant neoplasm of lung” (Fig. 5).

**Fig. 6:**
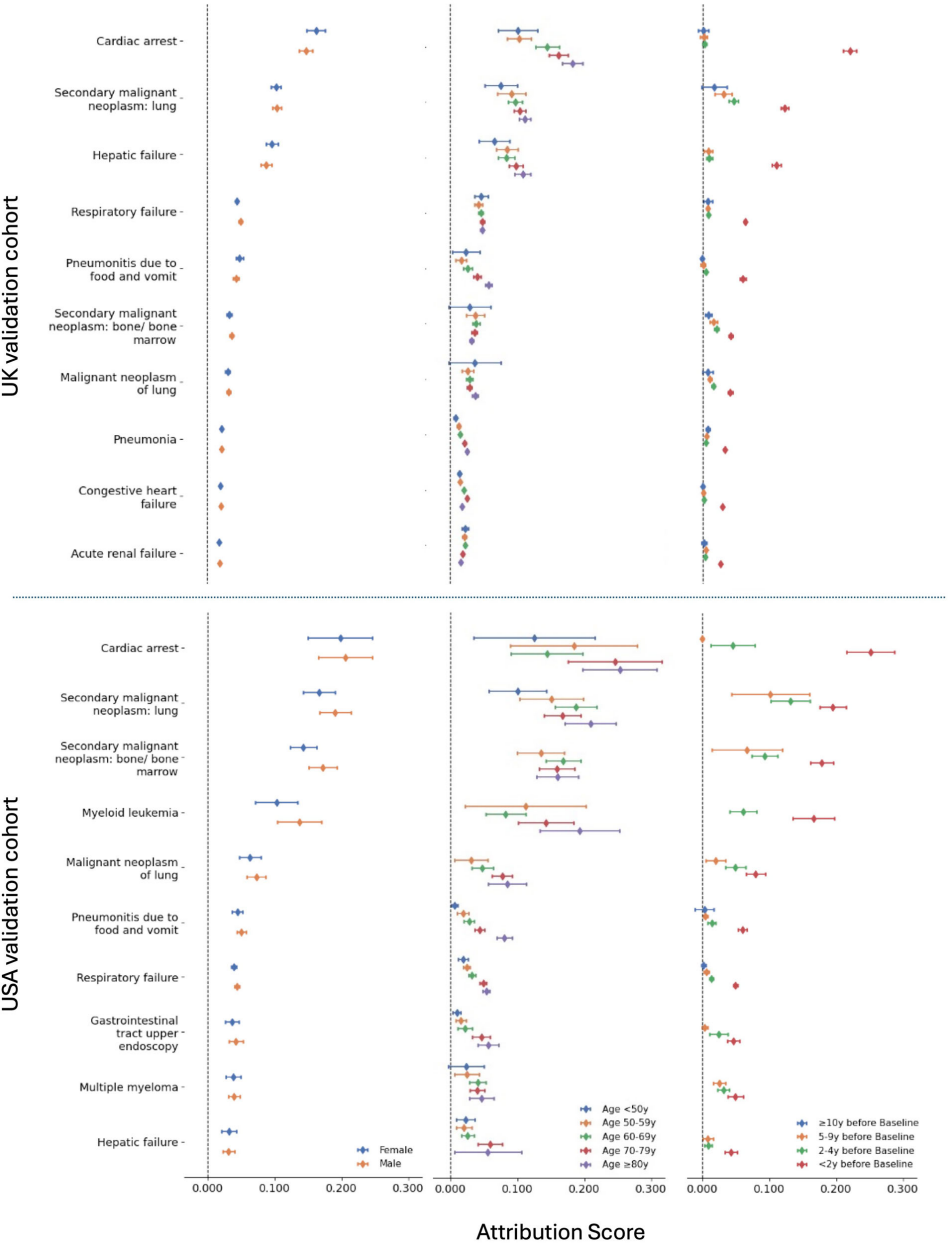
Stratified analyses of the top ten contributing encounters for mortality risk prediction on the UK and USA validation datasets. For all top ten contributing encounters, we investigated the contributions of the top ten contributing encounters in patient subgroups. Specifically, we investigated contribution patterns in both the UK (top) and the USA (bottom) validation cohorts stratified by sex, stratified by age at first encounter, and time between the first recording of encounter and baseline. Point estimates of contribution values and associated 95% CIs are presented for each encounter.

Sex-stratified analyses aligned with the main findings, demonstrating consistent patterns across male and female patients for the contributing encounters (Fig. 6). Age-stratified analyses showed encounter contributions generally increasing with age, particularly for patients aged 70 years and above (Fig. 6). Younger patients (aged 50–59 years) showed more modest attribution scores across all encounter types; middle-aged patients (60–69 years) demonstrated intermediate attribution scores, while older patients (70–79 years and ≥80 years) showed the highest attribution scores. This age-related gradient was most pronounced for “cardiac arrest”, “hepatic failure”, and respiratory conditions.

In time-to-baseline analysis (Fig. 6), most diseases showed higher contribution scores when closer to baseline, with diminishing association over time. Encounters occurring within one year of baseline consistently showed the highest attribution scores, typically 2–3 times higher than those occurring 5–10 years prior. However, cancers demonstrated a distinctive pattern, maintaining positive contributions even beyond 10 years before baseline, while other conditions showed more typical decay patterns over time. All cancer types, including “lung,” “bone/bone marrow,” and other secondary malignancies, showed attribution scores that remained strong contributors even beyond 10 years prior to baseline timepoint, suggesting persistent mortality associations long after initial diagnosis across both cohorts.

### Analyses of secondary outcomes on UK data

On CPRD, we additionally conducted analyses of several secondary outcomes: fatal and non-fatal cardiovascular outcomes (median follow-up of 5; IQI: [1, 16] months), cardiovascular-related mortality (9; IQI: [2, 28] months), and renal outcomes (6; IQI: [2, 20] months). Findings for the prediction of 36- (Figs. S9–S12 and Tables S2, S4, and S13–S15) and 12-month (Figs. S13–S15 and Table S8) risk of these secondary outcomes were overall similar to findings for all-cause mortality analyses. ICI-based calibration was reasonable for all outcomes, and calibration plots were generally sound. Smoothed calibration curves indicated minor under- and overestimation for certain outcomes, such as fatal and non-fatal cardiovascular events (details in Supplementary Results: Analyses of all outcomes on CPRD validation dataset).

## Discussion

As a model utilising solely routine EHR, TRisk demonstrated 10-20% higher *C*-index and 15–30% higher AUPRC than conventional models, with acceptable calibration across all outcomes at 12 and 36 months in the UK validation cohort. In impact analyses, the model reduced false positives and false negatives by up to 31% and 46%, respectively, compared to MAGGIC-EHR. Furthermore, TRisk transferred well from the UK to the USA EHR setting, with strong discrimination and calibration for mortality prediction. Finally, explainability analyses highlighted that the model captured validated predictors, providing face validity to AI feature extraction processes. Moreover, it revealed some underappreciated risk factors such as cancers, hepatic failure, and respiratory conditions across both cohorts.

The rising global burden of HF demands refined management^21^. Current state-of-the-art risk models for HF cohorts have several limitations that have impeded their widespread clinical adoption. Univariable risk tools like the New York Heart Association (NYHA) class or LVEF have shown limited prognostic value^22^. Additionally, multivariable risk tools (Table S16), such as the Seattle HF Model, are often tailored for specific HF patient groups, like those with reduced EF^4^. Few generally applicable models exist (e.g. MAGGIC-EHR)^3,8^; however, even they rely heavily on specialised imaging markers, indeed resource-intensive to procure. This creates barriers to implementation—particularly post-pandemic, where only 51% of hospitals meet the target of providing diagnostic echocardiography to 90% of HF patients^23^. Moreover, while these tests inform immediate care, their predictive value diminishes over time. This offers one explanation for why most models achieve modest discrimination (*C*-index < 0.8) for medium-term outcome prediction with accompanying poor sensitivity/PPV^7,13^. Our implementation of MAGGIC, amended for primary and secondary care EHR, demonstrated similar findings. As a result, HF management guidelines have been hesitant to integrate such models into care pathways.

TRisk, a model that accounts for complex patient journeys, demonstrated superior discrimination across four outcomes in the UK validation study of HF patients whilst maintaining calibration. Importantly, we found no evidence that TRisk exhibits biased predictions across key subgroups, including different sexes, age ranges, and baseline characteristics. This unbiased approach could be seminal for preventing disparities in HF management, maintaining equity in patient care and mitigating the perpetuation of existing inequalities^24^.

External validation across two distinct healthcare systems, CPRD (UK primary care) and MIMIC-IV (USA hospital and intensive care), highlights differences in patient populations, disease severity, and data recording practices that challenge direct validation and transfer learning. CPRD, captures longitudinal records from GPs and, with linkage to HES, hospital-admitted patient care, encompassing diagnoses, prescriptions, laboratory results, and procedures across the full spectrum of community and hospital care. This provides a population-level view of registered patients typically managed in outpatient or primary care settings with a comprehensive view of the HF journey^1,11^. In contrast, MIMIC-IV focuses on acutely hospitalised patients in tertiary and intensive care settings, with richer physiological and laboratory data from admissions but limited longitudinal follow-up after discharge unless externally linked^19^. Differences in coding systems, prescribing patterns, and healthcare access further contribute to heterogeneity between the datasets. Despite this heterogeneity, TRisk maintained strong discrimination and calibration, suggesting it captures generalisable clinical patterns rather than dataset-specific artefacts (as evident by the reproducibility of explainability analyses across the cohorts). Leveraging the large CPRD dataset for pre-training and fine-tuning on MIMIC-IV improved performance (by ~0.04 *C*-index), demonstrating successful transfer learning TRisk across multiple dimensions: from (1) larger to smaller cohort, (2) primary/secondary to inpatient/outpatient setting, and (3) UK to USA data. These findings indicate that TRisk can adapt across diverse clinical environments, though further validation and local recalibration remain seminal for deployment in new settings.

Data-driven AI models like TRisk offer a novel approach to predicting HF outcomes by capturing the holistic patient health journey. Unlike conventional models requiring extensive resources to identify specific predictors, TRisk automatically discovers important patterns from routine EHR data^14,16^. The model excels with complex patient data and perhaps counter-intuitively is designed for cohorts with numerous clinical visits, comorbidities, and procedures—precisely where conventional statistical models reach their limitation^8^. This represents a significant advance in risk assessment for patients with complex diseases at baseline.

An open question is how much of TRisk’s performance stems from the model architecture vs the intrinsic richness of the underlying data. While these factors are difficult to disentangle—since BEHRT, TRisk’s predecessor, was explicitly designed to leverage high-dimensional longitudinal EHR data—our results suggest that architecture contributes independently to performance. In previous work, BEHRT outperformed other recurrent and convolutional neural network models for longitudinal EHR using identical inputs without data imputation^14^. In this work, ablation analyses further demonstrate that TRisk’s architectural innovations improve performance over TRisk without concatenation-based embeddings (i.e. BEHRT-based survival modelling), indicating that both model design and data richness play complementary roles.

The explainability analyses further highlighted the importance of capturing complex patient journeys. While the model recognises established risk factors for mortality prediction, it identifies a broader range of conditions—particularly cancers, hepatic failure, and respiratory problems—as more significant predictors across both cohorts in line with recent research^2,25,26^. Additionally, temporal analysis of risk factors revealed that while recent clinical encounters generally carried greater predictive weight, cancer diagnoses maintained significant predictive value even ten years before baseline. This finding may reflect long-term sequelae of cancer itself (e.g. persistent systemic inflammation or immunologic dysregulation) or the cardiotoxic effects of prior cancer therapies (e.g. anthracyclines and radiotherapy), including accelerated vascular ageing^25,27^. Such potential mechanisms merit targeted future hypothesis discovery and validation studies in collaboration with cardio-oncologists. These insights directly explain the drivers of TRisk’s advantage over conventional models (e.g. MAGGIC), which omit longitudinal modelling of these and other identified predictors. Indeed, especially in complex patients with pre-existing diseases (e.g. HF, diabetes), these findings demonstrate the predictive value of capturing the full scope of a patient’s health trajectory—from historical conditions like cancer to recent clinical events—rather than relying solely on traditionally understood markers.

TRisk’s success promises significant clinical benefits throughout the HF care pathway without adding to the clinical workload by requiring additional information or specialised tests during consultations. At the population level, these risk scores can be used to audit HF service quality and assess the impact of service changes. At the individual level, TRisk can periodically estimate risk scores offline and flag patients at risk who may need further clinical attention, theoretically offering key advantages: better identification of low-risk patients could prioritise medication over transplantation, potentially reducing waitlists and healthcare costs, while accurate detection of high-risk cases could enable timely palliative care referrals, reducing hospitalisations and improving quality of life^28^.

Furthermore, TRisk has been validated for use across the spectrum of time-points following HF diagnosis via the selection of a random baseline. Although this methodological choice departs from conventional prognostic modelling practice, this design allows TRisk to learn from diverse points along the disease trajectory, enhancing generalisability and enabling dynamic risk estimation across varying follow-up intervals. This approach offers two key advantages. First, model generalisability: it trains the model to perform across the full spectrum of prognostication points rather than optimising for a single interval, producing a time-agnostic model robust to different clinical contexts. TRisk can indeed be utilised repeatedly, adapting to changes in patient health—whether from disease progression, treatment complications, or therapeutic interventions—by using updated EHR data to inform ongoing care decisions. Second, clinical adaptability: as EHR systems and digital health platforms (e.g. the NHS Single Patient Record initiative^29^) increasingly enable real-time risk estimation, models capable of valid predictions at arbitrary time points will be of greater practical value. This baseline selection strategy thus tests TRisk’s robustness in realistic settings. In future, if clinical stakeholders identify key time points for targeted prognostication, the TRisk model could be adapted to those intervals through targeted fine-tuning of the model and appropriate recalibration. Validated in both primary/secondary care (UK) and inpatient/outpatient care (USA) settings, TRisk can aid shared decision-making for HF management. While promising in several ways for improved HF management, we acknowledge that the present work does not model or evaluate intervention strategies. Instead, the improved prediction of medium-term outcomes (12- and 36-month mortality) demonstrates TRisk’s potential as a decision-support tool to be further explored in future, including through prospective evaluation.

In terms of study strengths, we comprehensively validated TRisk across two international datasets, assessing performance at 12 and 36 months as per guideline recommendations^13^. We analysed the model’s decision-making processes by examining influential medical encounters and discovered previously overlooked mortality risk factors. Finally, we demonstrated its versatility by testing on three additional outcomes using UK health records.

In terms of limitations, the MAGGIC model was not designed for use on routine EHR data. Specifically, LVEF measurements were fully absent while the NYHA classification variable suffered from substantial missingness (i.e. 96.6% of patients without NYHA classification). However, given the scarcity of risk prediction models for HF patients in routine EHR, we developed MAGGIC-EHR as a benchmark for comparison with TRisk. We also implemented MAGGIC-EHR + , which includes additional important predictors, to ensure fair comparison with AI approaches. Importantly, there is no evidence to suggest that our MAGGIC-EHR models prognosticate materially differently than the original model; in fact, predictive performance of our implementation aligns with previous MAGGIC validation studies (*C*-index: ~0.73; Table S16). Nevertheless, we acknowledge that the comparison between TRisk and MAGGIC-EHR is affected by the high level of missingness in key variables such as NYHA class and the complete absence of LVEF measures, which could lead to an artificially altered performance for MAGGIC-EHR. This limitation, in fact, highlights the practical challenges and reduced applicability of traditional risk scores when applied to routine EHR data settings.

TRisk requires access to complete EHR data and cannot be simplified to a basic scoring algorithm with a handful of predictors. While this increases implementation complexity compared to simpler regression models, robust tools exist to facilitate AI deployment in settings with limited computational capacity^30^. Future efforts could explore leveraging TRisk as an offline prognostication tool that periodically estimates risk scores, simultaneously improving audit of HF services via population-level analysis and delivering patient-level insights with individualised risk scores. This approach would integrate seamlessly into existing EHR systems without overwhelming healthcare professionals in demanding clinical environments.

To conclude, TRisk achieved superior prediction of clinical outcomes in HF patients using only routine EHR data, outperforming conventional models without sacrificing calibration. The model’s robust performance translated successfully to USA healthcare settings through transfer learning, demonstrating its potential for broader application. The explainability analyses revealed how risk profiles evolve over time, with both recent factors like hepatic failure and long-term influences like cancer driving prognostication. Utilising TRisk as an offline prognostication tool could improve audit of current and future HF services, along with refining care decisions at the individual level.

## Methods

### Data sources

For the UK data study, we used English data provided by the CPRD Aurum dataset with approval from the CPRD Independent Scientific Advisory Committee (protocol number: 20_095). CPRD covers approximately 20% of the UK population and is broadly representative of the UK population in terms of age, sex, and demographics. It includes detailed patients’ records, including demographics, diagnoses, prescribed treatments, and health-related lifestyle variables and provides linkage to Hospital Episode Statistics (HES) and Office for National Statistics (ONS) for data on hospital visits and mortality reports, respectively^18^.

For the USA data study, we used the Medical Information Mart for Intensive Care IV (MIMIC-IV) hospital admissions dataset covering admissions at Beth Israel Deaconess Medical Center in Massachusetts, USA, from 2008 to 2019^19^. This dataset contains health records from hospital admissions, including patient demographics, diagnoses, vital signs, laboratory test results, medications, and procedures. Additionally, the dataset provides linkages to the Centre’s outpatient records and Massachusetts State Registry of Vital Records and Statistics (RVRS) for data on outpatient visits and mortality records, respectively^19^.

Access to data from CPRD is subject to a full licence agreement containing detailed terms and conditions of use. Patient-level datasets can be extracted for researchers against specific study specifications, following protocol approval from the Independent Scientific Advisory Committee (ISAC). We used English data provided by the CPRD Aurum dataset with approval from the CPRD Independent Scientific Advisory Committee (protocol number: 20_095). The requirement for informed consent was waived by the ISAC because the CPRD data are pseudonymised. Ethical approval for the use of the MIMIC-IV database was obtained through completion of the required Collaborative Institutional Training Initiative (CITI) “Data or Specimens Only Research” course and acceptance of the PhysioNet data use agreement. For MIMIC-IV, informed consent was also not required as all data are de-identified.

### Primary outcome

The primary outcome of interest was all-cause mortality, with the date of outcome captured using linked ONS and RVRS mortality records for the UK and the USA, respectively.

### Validation strategy

We included data from 1418 contributing GP practices from England. We randomly assigned three-quarters (i.e. 1063) of practices to the derivation dataset, and the rest (i.e. 355) were allocated to a dataset for external validation. Derivation data was used for model fitting/training, while the validation data was used for validation of the models. For TRisk, to avoid overfitting in the training process, which is a concern with high-dimensional and AI parametric modelling, 5% of the derivation dataset was randomly selected to conduct end-of-epoch testing. This testing at the end of each epoch (i.e. one full iteration of training in which the model has trained on all patients selected for training) was used to ensure the model has not overfit on training data (i.e. ultimately aiming to avoid data “memorisation”). After the AI model training on 95% of patients in the derivation dataset was estimated to have converged, the AI model was evaluated on the validation dataset. All patients in the derivation dataset were used for the fitting of the conventional statistical modelling solutions.

### Cohort selection, index date, and follow-up period definition

We identified an open cohort of individuals between the ages of 40 and 90 years with their first HF diagnosis in the study period. The study period for each patient was defined as starting from the latest of the following: January 1st 2005, the patient’s 40th birthday, and 12 months after GP registration. It ended at the earliest of the following: date of outcome of interest (i.e. mortality), last collection of data from practice, patient’s last date in practice, or December 31st 2019. We excluded individuals who had an HF diagnosis before the study period. HF was identified by previously published phenotyping dictionaries for CPRD Aurum (Fig. S1)^21^.

For each eligible HF patient, we defined an eligibility window from the date of the first recorded HF diagnosis to the earliest of death, loss to follow-up, or the end of the study period. We then selected a single index date (i.e. baseline) by sampling one calendar day uniformly at random from this window, so that each patient contributed exactly one baseline assessment irrespective of subsequent survival time. No sampling step was conditioned on future survival or outcomes. This design yields a dynamic prediction setting in which the target quantity is the risk of death over a fixed horizon, conditional on the patient being alive and under follow-up at the index date. Patients who died on the same calendar day as their initial HF diagnosis and therefore had no post-diagnosis follow-up could not contribute an index date and are not part of this dynamic prediction population; model performance metrics should accordingly be interpreted for patients who survive long enough to be seen in follow-up. This approach captures HF patients across the spectrum of ages, calendar years, and disease stages, reflecting real-world clinical practice where patients are evaluated at different times along their care journey. By learning prognostic patterns from diverse timepoints, our model improves generalisability and supports dynamic, longitudinal risk estimation^31,32^. Patients were censored at the end of the study period.

### EHR pre-processing

For our AI modelling, we converted all diagnoses recorded by GPs from the Medcode diagnostic codes to the level 4 ICD-10 codes (i.e. 1 number following a decimal point like N29.9, J12.2, I50.1), ensuring uniformity in coding across hospital and general practice records. This conversion utilised a phenotyping dictionary from NHS Digital and SNOMED-CT. The medications were recorded in the format of the native CPRD product code. In which case, a British National Formulary (BNF) mapping was applicable, we translated the relevant product codes to their corresponding BNF codes, retaining only section-level codes (i.e. first four digits of the BNF coding format). For product codes that could not able to be mapped with the BNF’s mapping algorithm, we used NHS Digital maps, which mapped product codes to the appropriate virtual therapeutic moiety (VTM) codes, a vocabulary set for abstract representations of medication ingredients maintained and updated by the SNOMED-CT group. Consequently, our final medication code list included both BNF and VTM codes. In contrast, procedure codes, recorded as Office of Population Censuses and Surveys (OPCS) Classification of Interventions and Procedures version 4 codes, were maintained in their original format without undergoing any additional mapping or transformation. All codes that were prevalent in at least 0.1% of CPRD Aurum were used for modelling.

### AI modelling

TRisk is a deep learning Transformer model that adopts the BEHRT model for modelling longitudinal EHR and the survival ordinary differential equations network-based (SODEN) framework for survival prediction with additional regularisation procedures for better calibrated predictions compared to simpler AI models^14,33,34^. For input, TRisk considers a patient’s medical history up to baseline as a sequence of timestamped records^14,15^. The model incorporates all records from the following modalities in linked primary and secondary care records: 2,639 distinct diagnosis codes, 633 medication codes, and 852 procedure codes. Relative ordering and patient age at each encounter (e.g. diagnosis) are also provided to the model, thereby providing rich longitudinal annotations to the sequential stream of encounters in medical history up to index date (Fig. S2). The input provided to TRisk is as captured in routine EHR without imputation of missing values (model parameters in Table S1).

To understand the multi-component TRisk model (Fig. S2), an understanding of the BEHRT model is essential. BEHRT comprises of three primary elements: (1) an embedding layer that incorporates three layers of raw EHR data for modelling (see below for more details), (2) a transformer-based feature extractor, which extracts rich features from temporal EHR data utilising the multi-head self-attention mechanism, and (3) a binary prediction layer^14^. This layer uses a sigmoid activation function followed by a linear transformation of the latent patient representation vector to produce risk assessments (i.e. the likelihood of an outcome occurring) for an individual patient.

Regarding the (1) embeddings, the input embedding layers encompass encounter data, age, and positional encoding. The encounter layer includes diagnostic, medication, and any other datapoints from raw EHR (e.g. procedures), representing a patient’s medical history up to the baseline. The age layer specifies the exact age at the time of each encounter, calculated as a simple subtraction between the event date and estimated birth date; for the purposes of pseudonymisation, the CPRD supplies only the year of birth; therefore, the birth date is assumed to be July 1st of the stated birth year^18^. Positional encoding consists of pre-set embeddings that impart sequential context to the Transformer model. Pivotally, these positional embeddings denote visit number for a particular encounter; the encounters captured in the first visit will be given position encoding corresponding to visit 1, those for the second visit, the encoding to visit 2, and so on. The combined embeddings from all three features form the comprehensive high-dimensional vector representation of each encounter.

The TRisk model makes several changes to the underlying, BEHRT model to transform it into an accurate survival model^15^. First, the age embedding now explicitly includes age at baseline to more expressively capture the time of prediction. In this way, for a particular patient, prediction at age, 61 years, will be different from that at age, 65 years, and hence will be appropriately denoted differently. Also, in this way, baseline age is explicitly included as a variable for use in the prediction of the outcome of interest.

Second, the embedding layers are not summed like in previous iterations of BEHRT. Rather, the three separate embedding layers are concatenated using tensor concatenation operations and multiplied by a weight vector that transforms the space: 3•E → E (i.e. E being the space of one embedding layer; 3E is three stacked layers). Finally, a non-linear, hyperbolic tangent functional transformation is applied to the product to enable more expressive latent representation of the input raw EHR and ultimately, mitigate overfitting.

Third, TRisk utilises the SODEN framework for modelling. Instead of maximum likelihood estimation (MLE) frameworks that utilise costly integral calculations for model training on censored data, the SODEN framework alternatively poses MLE as a differential-equation constrained optimisation objective^34^. Furthermore, unlike previous proportional hazard frameworks that have not been shown to be theoretically robust for stochastic gradient descent (SGD) using mini-batching (i.e. random non-overlapping subsets of the derivation dataset), the SODEN framework alleviates many issues of established survival model frameworks that have trouble scaling to “big data”. Specifically, with ordinary differential equations modelling the time-to-event distribution, the framework for MLE on censored data becomes more flexible (i.e. absent of strong structural assumptions of the shape of the survival/hazard distribution) and more appropriately scalable (i.e. can be trained using SGD).

Finally, the TRisk objective or cost function utilises an Explicit Calibration (XCal) method for optimising for D-calibration, an omnibus measure of calibration, in addition to empirical loss^33^. With these updates, the TRisk model is theoretically more robust for scalable training on large-scale EHR data and ensures greater expressiveness and flexibility to ultimately ensure optimal discrimination and calibration.

The TRisk model, similar to the BEHRT model, utilises the prediction token (‘Predict token’) to serve as a dense patient representation vector for each patient. This vector, functioning as a dense feature vector, is then fed into the SODEN framework survival modelling network that conducts risk prediction for a particular outcome.

### Benchmark statistical model development

The MAGGIC-EHR model was adapted as a benchmark model for application on primary and secondary care EHR data^3^. All variables except for LVEF, which is not typically available in large-scale population-based EHR, were extracted from patient records and used in MAGGIC-EHR modelling. In lieu of the LVEF measurement, a categorical variable indicating HF subtype (i.e. reduced, preserved, and unknown LVEF), derived using published phenotyping codes, was incorporated into the model as a practical substitute^35^.

The raw variables extracted for MAGGIC-EHR modelling included sex, age, smoking status, diabetes, systolic blood pressure, creatinine, body mass index, NYHA Functional Class, chronic obstructive pulmonary disease, any use of beta-blockers, any use of ACE inhibitor/Angiotensin receptor blockers, incident HF diagnosis ≥ 18 months prior to baseline, and HF subtype (i.e. preserved, reduced, or unknown according to previously published method^35^). In addition, the age and systolic blood pressure interaction terms for the HF subtype were included in the model. For the extended MAGGIC-EHR model with additional predictors, referred to as the MAGGIC-EHR+ model, sodium level measurement, atrial fibrillation, stroke, myocardial infarction, history of percutaneous coronary intervention, and history of coronary artery bypass grafting procedure were extracted additionally. For both benchmark models, the predictors were captured from both primary care and HES records. The average of records for systolic blood pressure, creatinine, body mass index, and sodium that were collected in the 36 months leading up to baseline were used as baseline variables for modelling. Last known smoking status in the 36 months leading up to baseline was used as baseline smoking status.

Missing values for systolic blood pressure, smoking status, body mass index, creatinine, and NYHA Functional Class were imputed for the MAGGIC-EHR model. Separate imputation was conducted for the MAGGIC-EHR+ model, accounting for missing sodium measurements. Procedurally, for the imputation of all-cause mortality prediction MAGGIC-EHR and MAGGIC-EHR+ models, all variables for each model were included for imputation, along with the Nelson-Aalen estimate of baseline cumulative hazards for the outcome of mortality. Imputations were conducted on each derivation and validation dataset of our cohort separately using the “mice: Multivariate Imputation by Chained Equations” package in R^24^. Following the extraction of derivation and validation datasets, the statistical models were fit on each of the five imputed derivation datasets with appropriate predictors (i.e. 5 MAGGIC-EHR/MAGGIC-EHR+ models) for the prediction of the primary outcome, all-cause mortality. The five Cox models were pooled using Rubin’s rules and evaluated on the 5 imputed validation datasets. The predictions were also appropriately pooled using extensions of Rubin’s rules (i.e. complementary log-log transformations) and utilised for downstream analysis.

### Performance analysis on UK validation data

We evaluated models’ performance on the UK validation dataset using multiple metrics. For discrimination, we assessed the concordance index (*C*-index) and area under the precision-recall curve (AUPRC). The *C*-index measures the model’s ability to correctly rank-order patients by risk—that is, the probability that, for any randomly selected pair, the patient with the higher predicted risk experiences the event sooner (with a *C*-index of 0.5 indicating “at-random” performance). The AUPRC captures the trade-off between precision and recall, providing a more informative evaluation of performance on the positive class, particularly in the context of class imbalance.

Model calibration was assessed graphically, by comparing the agreement between the observed and predicted risk using a smoothed calibration curve fit using restricted cubic splines with three knots, and quantitatively by calculating the integrated calibration index (ICI) metric^36^. Decision curve analysis (accounting for censoring) assessed the balance between true positives (correctly identified mortality) and false positives across the risk spectrum^37^. For all analyses, individual patient risk scores were derived by estimating survival probabilities at the 36-month timepoint.

Additionally, we conducted discrimination analyses in subgroups defined by sex, age, baseline systolic blood pressure, HF subtype (i.e. preserved or reduced LVEF as identified by the algorithm introduced previously^35^), baseline disease status, and baseline medication use.

To ensure fairer comparison with TRisk, we implemented MAGGIC-EHR with an extended predictor set (denoted henceforth as MAGGIC-EHR+) that included additional established risk factors: serum sodium concentration, as well as history of atrial fibrillation, stroke, myocardial infarction, percutaneous coronary intervention, and coronary artery bypass grafting procedure^2^.

To evaluate the incremental contribution of key architectural components, we conducted ablation analyses on TRisk using the UK CPRD cohort for 36-month all-cause mortality prediction. The following model variants were assessed: (1) TRisk without Explicit Calibration [XCal]: The explicit calibration regularisation term was removed during optimisation to assess its effect on calibration and discrimination. (2) TRisk without concatenated Embeddings: The linear mapping in TRisk’s embedding layer was replaced with the original BEHRT-style token concatenation (summation of diagnosis, position, segment, and age embeddings). (3) TRisk without Explicit Calibration [XCal] and Concatenated Embeddings: Both modifications were removed to examine potential interaction effects. All models were trained and evaluated under identical conditions and hyperparameters to the reference TRisk model. Finally, to elucidate the utility of deep learning more generally, we compared against a random survival forest model with the same 2639 distinct diagnosis codes, 633 medication codes, and 852 procedure codes used for TRisk modelling encoded as one-hot embeddings. For all models, performance was assessed using the concordance index (*C*-index) for discrimination, the area under the precision-recall curve (AUPRC), and the integrated calibration index (ICI) for calibration.

Calibration and net benefit analyses for 12-month prediction were also conducted for all models to assess model performance at different follow-up durations^2^.

Furthermore, we conducted an impact analysis to evaluate true positive and negative capture at the 25%, 50%, and 75% decision thresholds of the TRisk and MAGGIC-EHR models on UK validation data in line with research recommendations by HF clinical guidelines^13^.

### Analyses of USA data

We conducted external validation of TRisk for 36-month all-cause mortality prediction in the MIMIC-IV USA dataset as an entirely independent cohort^19^. Following a similar cohort selection used on CPRD, we randomly allocated 60% of the identified HF patient cohort to the validation dataset, while the remaining 40% formed the fine-tuning dataset.

On a random selection of 60% of the cohort, we conducted external validation of the TRisk model, henceforth referred to as the MIMIC-IV external validation dataset. We used the remaining 40% of the identified cohort for transfer learning and fine-tuning experiments, henceforth referred to as the MIMIC-IV fine-tuning dataset. Within the 40% cut of the dataset, for experiments involving the TRisk model fitting/training, 10% of the dataset was randomly selected to conduct end-of-epoch testing similar to CPRD. All models were evaluated on the MIMIC-IV external validation dataset.

We evaluated three different models on the validation dataset of MIMIC-IV: (1) TRisk trained on CPRD, (2) TRisk trained “from scratch” on the fine-tuning dataset, and (3) TRisk trained on CPRD, and transferred and further fine-tuned on the fine-tuning dataset.

As TRisk requires diagnosis, medication, and procedure records to be encoded in accepted vocabularies (e.g. ICD-10 for diagnosis records), we conducted the mapping for the MIMIC-IV dataset. Specifically, since the TRisk needed to be successfully transferred from CPRD to MIMIC-IV, raw records from MIMIC-IV needed to be mapped to the same vocabulary sets: (1) ICD-10 for diagnoses, (2) mixed, BNF and VTM for medications, and (3) OPCS for procedures. For diagnosis records, the MIMIC-IV dataset provides records in a mixture of ICD-9 and ICD-10 codes. ICD-9 codes were mapped to ICD-10 codes using established maps provided by the public code repository, “MIMIC-IV-Data-Pipeline”, the MIMIC-IV data processing pipeline developed by Gupta et al. (https://github.com/healthylaife/MIMIC-IV-Data-Pipeline). For medication records, we used SNOMED CT maps provided by Athena, the Observational Health Data Sciences and Informatics (OHDSI) vocabularies repository to map the National Drug Code (i.e. drug encoding vocabulary in the USA) to BNF and VTM DM + D (i.e. SNOMED) codes. For the procedures, encoded in ICD-9 and ICD-10 Procedure Coding System (PCS), we mapped them to SNOMED universal procedure codes and finally OPCS using Athena vocabulary mapping libraries^38^.

For all models, we conducted discrimination, calibration, and decision curve analyses. Lastly, all analyses were replicated for 12-month mortality prediction.

### Explainability analyses of TRisk

To understand TRisk’s decision-making processes, we used the integrated gradients method to analyse which medical history encounters most influenced risk score calculation^20^. This method measures how each encounter (e.g. diagnosis, prescription, procedure) in a patient’s medical history contributes to the risk score; to capture each encounter’s population-level importance, we then averaged these contribution scores across the cohort. In this work, we adapted the integrated gradients method to work within the SODEN framework, calculating how each patient encounter influences the model’s output. For repeated encounters (e.g. multiple diagnoses of the same condition or multiple prescriptions of the same medication), we used the highest contribution value across all instances. We focused our analysis on encounters that appeared in at least 1% of patients to ensure statistical reliability.

Our population analysis consisted of two stages: validating TRisk’s ability to detect established clinical risk factors^2,3^, then determining which encounters it independently identified as most significant. We analysed these patterns across sex, age, and time from first encounter to baseline in the UK validation cohort. In age-based analyses, for each encounter, we grouped patients into subgroups based on the age of the first recording of the particular encounter. Similarly, for the analyses of time between the recording of the encounter and baseline, we grouped patients into subgroups based on the first (as opposed to any other subsequent recording) recording of encounter and baseline. To ensure robustness of our explainability discoveries, we conducted parallel validation on the USA validation cohort.

### Analyses of secondary outcomes on UK data

Finally, we conducted supplementary analyses to assess the utility of TRisk for the prediction of outcomes other than all-cause mortality. These outcomes included: (1) fatal and non-fatal cardiovascular event prediction (i.e. composite of ischaemic heart disease, myocardial infarction, transient ischaemic attack, and stroke), (2) cardiovascular-related mortality prediction, and (3) renal outcomes prediction (i.e. composite of chronic kidney and end-stage renal diseases). Outcomes were identified using both previously published and our own curated code dictionaries^21,39^. Similar to all-cause mortality prediction analysis, we evaluated all models on the UK validation data and discrimination, calibration and net benefit for outcome investigations were assessed; analyses were replicated for 12-month prediction of all outcomes^2^.

For TRisk, the model was derived in the same way as for all-cause mortality prediction. For the benchmark statistical models, the same procedures for model fitting and evaluation on validation datasets were repeated for the analyses of the secondary outcomes. Interestingly, it must be noted that the original MAGGIC model derivation study has solely investigated 12- and 36-month all-cause mortality^3^. However, while the MAGGIC risk score was not derived for secondary outcome investigations, validation studies have indeed been conducted for other outcomes such as CV-related mortality (more details in Table S16)^8^.

### Reporting guidelines

For this research, we followed the TRIPOD-AI reporting guidelines to ensure transparent and rigorous reporting of our AI-based predictive modelling study^40^.

## Supplementary information

NPJ-DM_2ndRound_Revision_Supplementary_TRisk_HF

## Data Availability

CPRD data are available to researchers through licensing agreements following protocol approval by the Independent Scientific Advisory Committee. Data are not publicly available due to licensing restrictions. Access and sharing policies are detailed on the CPRD website. The MIMIC-IV database is freely available for research use upon completion of the Collaborative Institutional Training Initiative (CITI) “Data or Specimens Only Research” course and signing of the data use agreement, with access obtained through the PhysioNet organisation and website.

## Code availability

The TRisk model codebase is available at: https://innovation.ox.ac.uk/technologies-available/technology-licensing/. The data pre-processing is similar to that of the BEHRT modelling framework. The data pre-processing code, as well as the BEHRT model code is available here: https://github.com/deepmedicine/BEHRT/tree/master. Pre-trained model weights are available upon request of the corresponding authors. Interested researchers are welcome to contact corresponding authors directly via the corresponding email address provided to obtain weights for research purposes.
