# Supplementary material for: A transformer-based survival model for prediction of all-cause mortality in patients with heart failure: a multi-cohort study
Source: NPJ Digit Med. 2026 Jan 8;9:118. doi: 10.1038/s41746-025-02296-5 (PMC12868603; doi:10.1038/s41746-025-02296-5)
Supplement: Supplementary file 1 — NPJ-DM_2ndRound_Revision_Supplementary_TRisk_HF [file 41746_2025_2296_MOESM1_ESM.pdf]

Supplementary Material

A Transformer-based survival model for prediction of all-cause mortality in patients with heart failure: a multi-cohort study

Shishir Rao[1], Nouman Ahmed[1], Gholamreza Salimi-Khorshidi[1], Christopher Yau[1,2], Huimin Su[3], Nathalie Conrad[1,3], Folkert W Asselbergs[2,4,5], Mark Woodward[6,7], Rod Jackson[8], John GF Cleland[9], Kazem Rahimi[1*]

[1] Nuffield Department of Women's & Reproductive Health, University of Oxford, Oxford, United Kingdom
[2] Health Data Research UK, London, UK
[3] Department of Cardiovascular Sciences, Katholieke Universiteit Leuven, Belgium
[4] Amsterdam University Medical Center, Department of Cardiology, University of Amsterdam, Amsterdam, The Netherlands.
[5] Institute of Health Informatics, University College London, London, UK
[6] The George Institute for Global Health, University of New South Wales, Newtown, NSW, Australia
[7] The George Institute for Global Health, Imperial College London, London, United Kingdom
[8] School of Population Health, Faculty of Medical and Health Sciences, University of Auckland, Auckland, New Zealand
[9] British Heart Foundation Centre of Research Excellence, School of Cardiovascular and Metabolic Health, University of Glasgow, Glasgow, UK

Table of Contents

Supplementary Results ... 3
Supplementary Results: Ablation analyses ... 3
Supplementary Results: Analyses of all outcomes on CPRD validation dataset ... 3
Supplementary Results: Impact analyses on CPRD validation dataset ... 3
Supplementary Results: Analyses on MIMIC-IV validation dataset ... 4
References ... 5
Supplementary Figures ... 6
Figure S1. Cohort selection flowchart for Clinical Practice Research Datalink (CPRD) Aurum dataset. 6
Figure S2. TRisk input space for a hypothetical patient's medical history. ... 7
Figure S3. Discriminative performance of models for 36-month risk prediction of all-cause mortality on UK validation data. ... 8
Figure S4. Calibration curves, distribution of predicted risk of models, decision curve analyses for 36-month all-cause mortality across all models on UK validation data. ... 9
Figure S5. Calibration curves, distribution of predicted risk of models, decision curve analyses for 12-month all-cause mortality across all models on UK validation data. ... 10
Figure S6. Cohort selection for MIMIC-IV hospital admissions validation dataset. ... 11
Figure S7. Calibration curves, distribution of predicted risk of models, and decision curve analysis for 12-month all-cause mortality risk prediction on USA validation data. ... 12
Figure S8. Average contribution for validated risk factors contributing to mortality risk prediction on UK and USA validation cohort datasets. ... 13
Figure S9. Discriminative performance of models for 36-month risk prediction of various outcomes on UK validation data. ... 14
Figure S10. Calibration curves, distribution of predicted risk of models, decision curve analyses 36-month non-fatal/fatal cardiovascular event prediction on UK validation data. ... 15
Figure S11. Calibration curves, distribution of predicted risk of models, decision curve analyses 36-month CV-related mortality prediction on UK validation data. ... 16
Figure S12. Calibration curves, distribution of predicted risk of models, decision curve analyses 36-month renal outcomes prediction on UK validation data. ... 17
Figure S13. Calibration curves, distribution of predicted risk of models, decision curve analyses 12-month non-fatal/fatal cardiovascular event prediction on UK validation data. ... 18
Figure S14. Calibration curves, distribution of predicted risk of models, decision curve analyses 12-month CV-related mortality prediction on UK validation data. ... 19

Figure S15. Calibration curves, distribution of predicted risk of models, decision curve analyses 12-month renal outcomes prediction on UK validation data. 20
Supplementary Tables 22
Table S1. Model hyperparameters and other settings for TRisk 22
Table S2. Area under the precision-recall curve (AUPRC) metrics for all models for all 36-month risk prediction investigations on UK validation data. 23
Table S3. Subgroup discrimination analysis for 36-month all-cause mortality prediction investigations on UK validation data 24
Table S4. Integrated calibration index (ICI) for various 36-month risk prediction investigations across all models on UK validation data 25
Table S5. Ablation analyses on TRisk model: prognostication of 36-month all-cause mortality in UK dataset 26
Table S6. Random survival forest modelling hyperparameter search 27
Table S7. Random survival forest modelling: prognostication of 36-month all-cause mortality in UK dataset 28
Table S8. Integrated calibration index (ICI) for various outcome 12-month risk prediction investigations across all models on UK validation data 29
Table S9. Impact analyses at the various decision thresholds for 12- and 36-month all-cause mortality prediction on UK validation data 30
Table S10. Population characteristics of the MIMIC-IV cohort 31
Table S11. Area under the precision-recall curve (AUPRC) metrics for all models for all 12- and 36-month risk prediction of all-cause mortality prediction on MIMIC-IV validation data 32
Table S12. Integrated calibration index (ICI) for 12- and 36-month risk prediction of all-cause mortality on MIMIC-IV validation data across all models 33
Table S13. Subgroup discrimination analysis for 36-month cardiovascular-related mortality prediction investigations on UK validation data 34
Table S14. Subgroup discrimination analysis for 36-month non-fatal/fatal cardiovascular event prediction investigations on UK validation data 35
Table S15. Subgroup discrimination analysis for 36-month renal outcomes prediction investigations on UK validation data 36
Table S16. Multivariable risk models for patients with heart failure 37

Supplementary Results

Supplementary Results: Ablation analyses

Ablation results are presented in **Table S5**. The reference TRisk model achieved a C-index of 0.845 (95% CI: 0.841, 0.849), AUPRC = 0.797, and ICI = 0.042. Removing explicit calibration slightly reduced discrimination (C-index = 0.843 [0.838, 0.847]) and degraded calibration (ICI = 0.056). Replacing the linear embedding mapping with simple concatenation produced a larger drop in discrimination (C-index = 0.828 [0.824, 0.833]) and slightly worsened calibration (ICI = 0.043). Removing both modelling features yielded similar results (C-index = 0.828 [0.823, 0.832]; ICI = 0.054). These findings confirm that explicit calibration regularisation improves calibration reliability, while the enhanced embedding formulation contributes to improved discrimination. Overall, the analyses show that each architectural refinement in TRisk offers incremental but meaningful performance gains over the BEHRT-style baseline.

Supplementary Results: Analyses of all outcomes on CPRD validation dataset

For all-cause mortality prediction, TRisk demonstrated significantly higher C-index and AUPRC as compared to MAGGIC-EHR class of models **(Figure S3; Table S2)**. Statistical models were similar across both metrics (**Figure S3; Table S2**). In terms of predictive distribution, the AI approach clearly demonstrated two peaks in the lowest and highest ends of the spectrum of predicted risk implying more nuanced stratification of risk as compared to the MAGGIC-EHR class of models (**Figures 2B, S4B)**. Decision curve analysis showed that TRisk provided significantly greater net benefit than both MAGGIC-EHR and MAGGIC-EHR+ models (**Figure S4C**) across the spectrum of clinically relevant thresholds (0.0-0.6). The subgroup discrimination analyses demonstrated that the deviation from overall cohort discrimination was mitigated for TRisk as compared to benchmark statistical modelling solutions (**Table S3**). 12-month model performance captured similar trends found in 36-month analyses (**Figure S5; Table S8**).

In analyses of secondary outcomes, overall and subgroup discrimination, calibration, and decision curve trends for models predicting both 36- (**Figures S9-S12; Table S2, S4, S13-S15**) and 12-month (**Figures S13-S15; Table S8**) risk were generally similar to trends captured in analysis of the main outcome. ICI calibration measures were reasonable for all outcomes; however, for certain outcomes (e.g., fatal and non-fatal cardiovascular events), calibration plots showed minor deviations from the line of parity.

Significant advancements in AI modelling were key for securing TRisk's gains in discrimination whilst remaining well-calibrated. TRisk with the SODEN framework and the enhanced EHR data representation layer outperformed conventional models. The SODEN framework offered more flexible modelling of the outcome space as compared to the proportional hazards framework (i.e., the framework used in Cox models and DeepSurv models), crucial for complex cohorts such as those with HF.[1] Additionally, TRisk's nuanced non-linear embedding module enabled extraction of more informative and richer features from minimally processed EHR data than predecessor models. In terms of calibration, TRisk demonstrated acceptable calibration, as evidenced by visual and ICI metrics. In summary, the fusion of the (1) revised EHR representation layer, (2) Transformer-based SODEN framework, and (3) explicit calibration regularisation scheme for improved calibration underscores the novelty and effectiveness of TRisk.

Supplementary Results: Impact analyses on CPRD validation dataset

In impact analysis (**Figure 3; Table S9**), the benchmark MAGGIC-EHR model predicted ≥ 50% mortality rate in the 12 months following baseline for 13,042 of the 31,634 who actually died (i.e., sensitivity: 0.412) with 9,870 false alarms (i.e., PPV: 0.569). On the other hand, TRisk captured 1.7 times more correct cases with 21,586 accurate predicted cases (i.e., recall: 0.682) and predicted 699 fewer false alarms (i.e., PPV: 0.702).

Similarly, evaluating models with ≥ 50% predicted mortality rate in the 36 months following baseline, TRisk outperformed the benchmark approach with 0.105 and 0.059 greater PPV and sensitivity respectively. Importantly, TRisk demonstrated improvements in PPV and sensitivity metrics all whilst identifying 5,310 fewer at-risk patients than MAGGIC-EHR. Ultimately, TRisk enabled more accurate prediction with both fewer false positives and negatives as compared to benchmark MAGGIC-EHR approach.

We further examined additional thresholds of 0.25 and 0.75. TRisk consistently demonstrated superior practical performance compared with MAGGIC-EHR on the UK CPRD validation cohort (**Table S9**). At lower thresholds (e.g., 0.25), both models identify larger patient groups as "high risk," but TRisk attains higher positive predictive values (PPV), indicating improved precision. However, at this threshold, there was slightly poorer sensitivity as compared to the MAGGIC-EHR model. At the 50% threshold (reported in the manuscript), TRisk identified substantially more true positives with fewer false alarms than MAGGIC-EHR (higher PPV and sensitivity while flagging fewer patients overall). At the higher, more conservative threshold (0.75), both models identify smaller high-risk subsets; here TRisk again maintains relatively higher PPV and sensitivity, suggesting better discrimination even when targeting only the most extreme predicted risks. In gist, the TRisk model applied at thresholds ≥0.25 (e.g., 0.5, 0.75) would be more preferable as it shows both improved sensitivity and precision as compared to benchmark modelling.

Supplementary Results: Analyses on MIMIC-IV validation dataset

In analyses of the validation dataset for 36-month mortality prediction, the transfer learning variant of TRisk demonstrated superior C-index of 0.802 (0.789, 0.816) compared to other TRisk modelling strategies with similar superiority in AUPRC (**Figure 4; Table S9**). TRisk without prior trained weights (i.e., randomly initialised), solely trained on MIMIC-IV fine-tuning data performed akin to a random, "coin-flip" risk model (i.e., ~0.5 C-index). In terms of calibration, while externally validated TRisk suffered from underestimation of risk at thresholds upwards of 0.4, the transfer learned TRisk was found to be better calibrated across the full spectrum of risk (**Figure S8; Table S11**) with better stratification of high-risk individuals as evidenced by a peak in the high end of the risk spectrum (**Figure 4C**). Decision curve analyses demonstrated that the transfer learning TRisk variant provided greatest net benefit across all relevant thresholds (**Figure 4D**). All findings were preserved in analyses of 12-month mortality prediction (**Figure S7; Tables S9, S11**).

References

1. Tang, W., Ma, J., Mei, Q. & Zhu, J. SODEN: A Scalable Continuous-Time Survival Model through Ordinary Differential Equation Networks. http://arxiv.org/abs/2008.08637 (2020).
2. Levy, W. C., Mozaffarian, D., Linker, D. T., Sutradhar, S. C., Anker, S. D., Cropp, A. B., Anand, I., Maggioni, A., Burton, P., Sullivan, M. D., Pitt, B., Poole-Wilson, P. A., Mann, D. L. & Packer, M. The Seattle Heart Failure Model: prediction of survival in heart failure. *Circulation* 113, 1424–1433 (2006).
3. Allen, L. A., Matlock, D. D., Shetterly, S. M., Xu, S., Levy, W. C., Portalupi, L. B., McIlvennan, C. K., Gurwitz, J. H., Johnson, E. S., Smith, D. H. & Magid, D. J. Use of Risk Models to Predict Death in the Next Year Among Individual Ambulatory Patients With Heart Failure. *JAMA Cardiol* 2, 435–441 (2017).
4. Canepa, M., Fonseca, C., Chioncel, O., Laroche, C., Crespo-Leiro, M., Coats, A., Mebazaa, A., Piepoli, M. F., Tavazzi, L., Maggioni, A. P., Anker, S., Filippatos, G., Ferrari, R., Amir, O., Dahlström, U., Delgado Jimenez, J. F., Drozdz, J., Erglis, A., Fazlibegovic, E., Fruhwald, F., Gatzov, P., et al. Performance of Prognostic Risk Scores in Chronic Heart Failure Patients Enrolled in the European Society of Cardiology Heart Failure Long-Term Registry. *JACC Heart Fail* 6, 452–462 (2018).
5. Pocock, S. J., Ariti, C. A., McMurray, J. J. V., Maggioni, A., Køber, L., Squire, I. B., Swedberg, K., Dobson, J., Poppe, K. K., Whalley, G. A. & Doughty, R. N. Predicting survival in heart failure: a risk score based on 39 372 patients from 30 studies. *Eur Heart J* 34, 1404–1413 (2013).
6. McDowell, K., Kondo, T., Talebi, A., Teh, K., Bachus, E., de Boer, R. A., Campbell, R. T., Claggett, B., Desai, A. S., Docherty, K. F., Hernandez, A. F., Inzucchi, S. E., Kosiborod, M. N., Lam, C. S. P., Martinez, F., Simpson, J., Vaduganathan, M., Jhund, P. S., Solomon, S. D. & McMurray, J. J. V. Prognostic Models for Mortality and Morbidity in Heart Failure With Preserved Ejection Fraction. *JAMA Cardiol* 9, 457–465 (2024) doi:10.1001/jamacardio.2024.0284.
7. Khanam, S. S., Choi, E., Son, J. W., Lee, J. W., Youn, Y. J., Yoon, J., Lee, S. H., Kim, J. Y., Ahn, S. G., Ahn, M. S., Kang, S. M., Baek, S. H., Jeon, E. S., Kim, J. J., Cho, M. C., Chae, S. C., Oh, B. H., Choi, D. J. & Yoo, B. S. Validation of the MAGGIC (Meta-Analysis Global Group In Chronic Heart Failure) heart failure risk score and the effect of adding natriuretic peptide for predicting mortality after discharge in hospitalized patients with heart failure. *PLoS One* 28 13 (2018).
8. Sartipy, U., Dahlström, U., Edner, M. & Lund, L. H. Predicting survival in heart failure: Validation of the MAGGIC heart failure risk score in 51 043 patients from the Swedish Heart Failure Registry. *Eur J Heart Fail* 16, 173–179 (2014).

Supplementary Figures

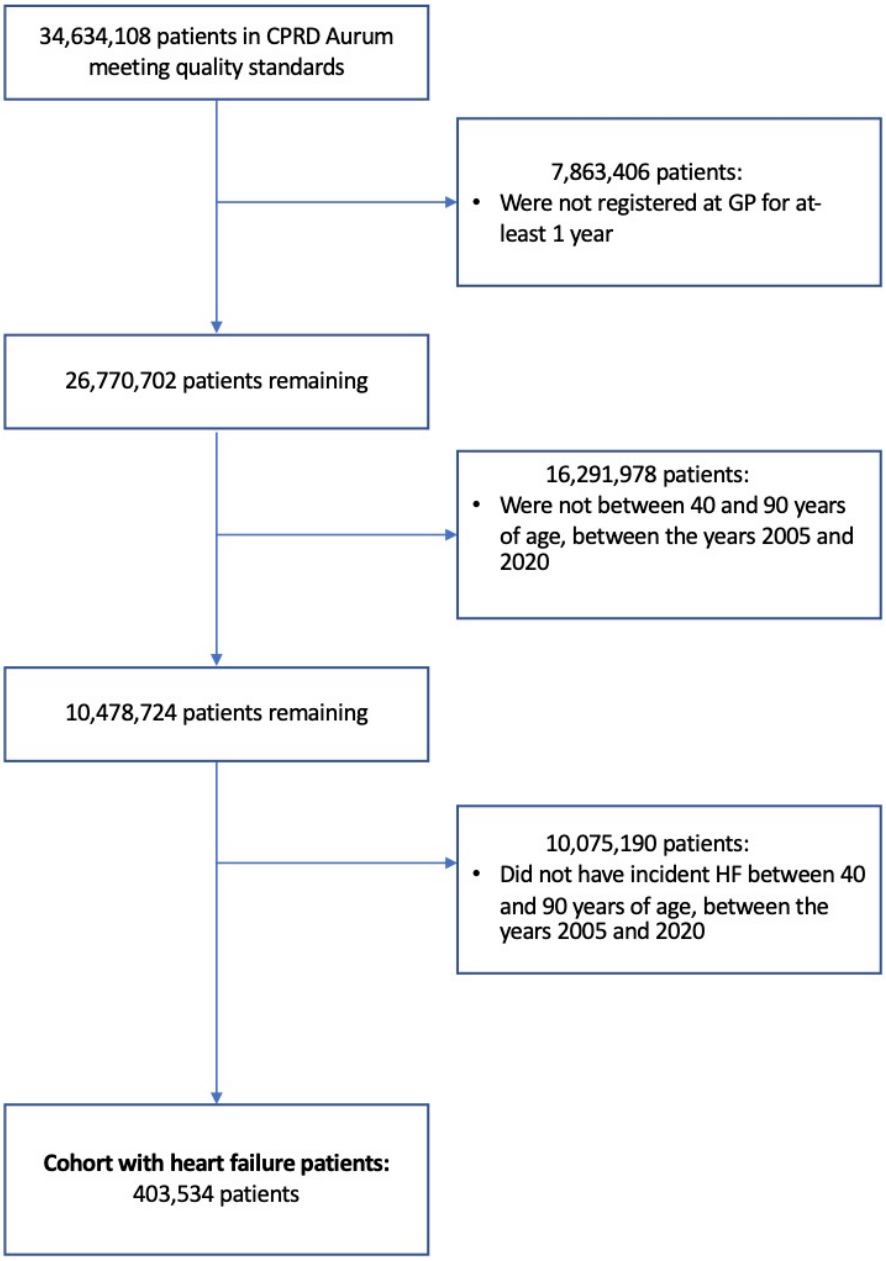

Figure S1. Cohort selection flowchart for Clinical Practice Research Datalink (CPRD) Aurum dataset.

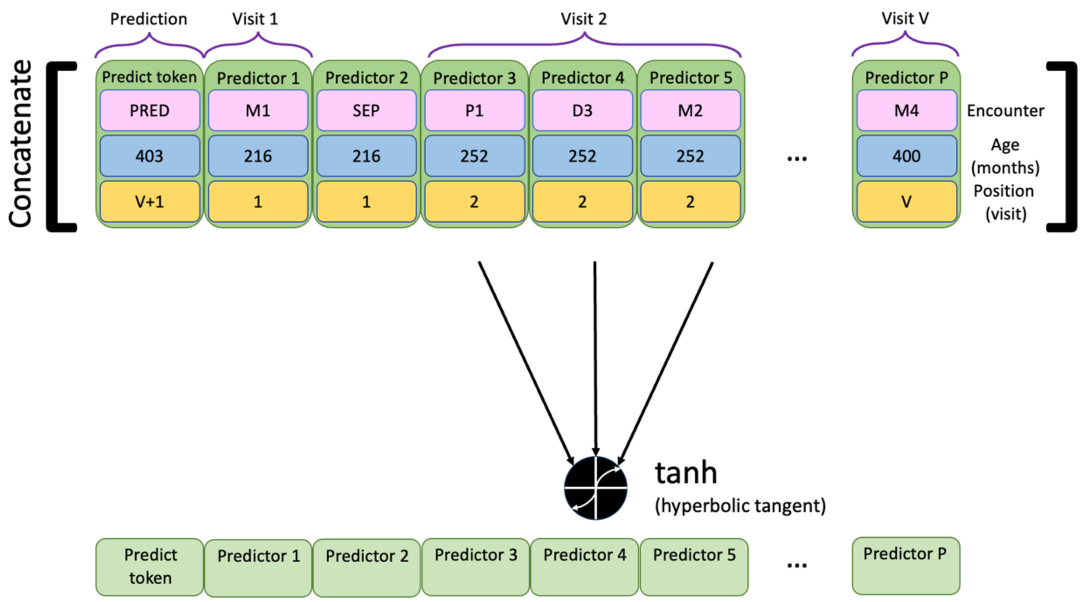

Figure S2. TRisk input space for a hypothetical patient's medical history.

For a hypothetical patient, the figure presents a sequence of medical history records represented by a group of three variables. Each record is comprised of an encounter (e.g., "M1" representing a hypothetical medication), the age at which the encounter was recorded in months (e.g., "216" months), and the visit number (e.g., visit "1"). These three components of the raw electronic health record data are represented by embeddings inputted into the model. The first character of the encounter code, D, M, and P represent diagnosis, medication, and procedure records respectively represented by a code in this figure. The number following the letter illustrates a hypothetical code of that modality type (e.g., 'D3' might represent code "I51.2" in ICD-10 encoding). "SEP" represents a separation character given to the model to split up the data between visits. Lastly, the "PRED" token (i.e., "predict token") is a special token that is used for prediction of the outcome (e.g., all-cause mortality outcome prediction). This special token has the age at baseline (e.g., "403" months at baseline for this hypothetical patient). As this is the visit that will happen following the final visit (i.e., visit number "V"), the visit for the predict token is appropriately enumerated as visit number "V+1". This predict token is inputted as displayed and following further transformation by Transformer architecture layers, the corresponding output state token will be used as a condensed latent patient representation layer for input into the ordinary differential equation-based survival prediction network layers.

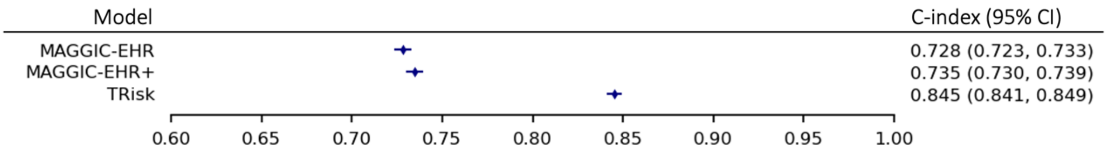

Figure S3. Discriminative performance of models for 36-month risk prediction of all-cause mortality on UK validation data.

Discrimination is provided in this forest plot as assessed by C-index with 95% confidence intervals (CI) for various outcome investigations at 36-month timepoint.

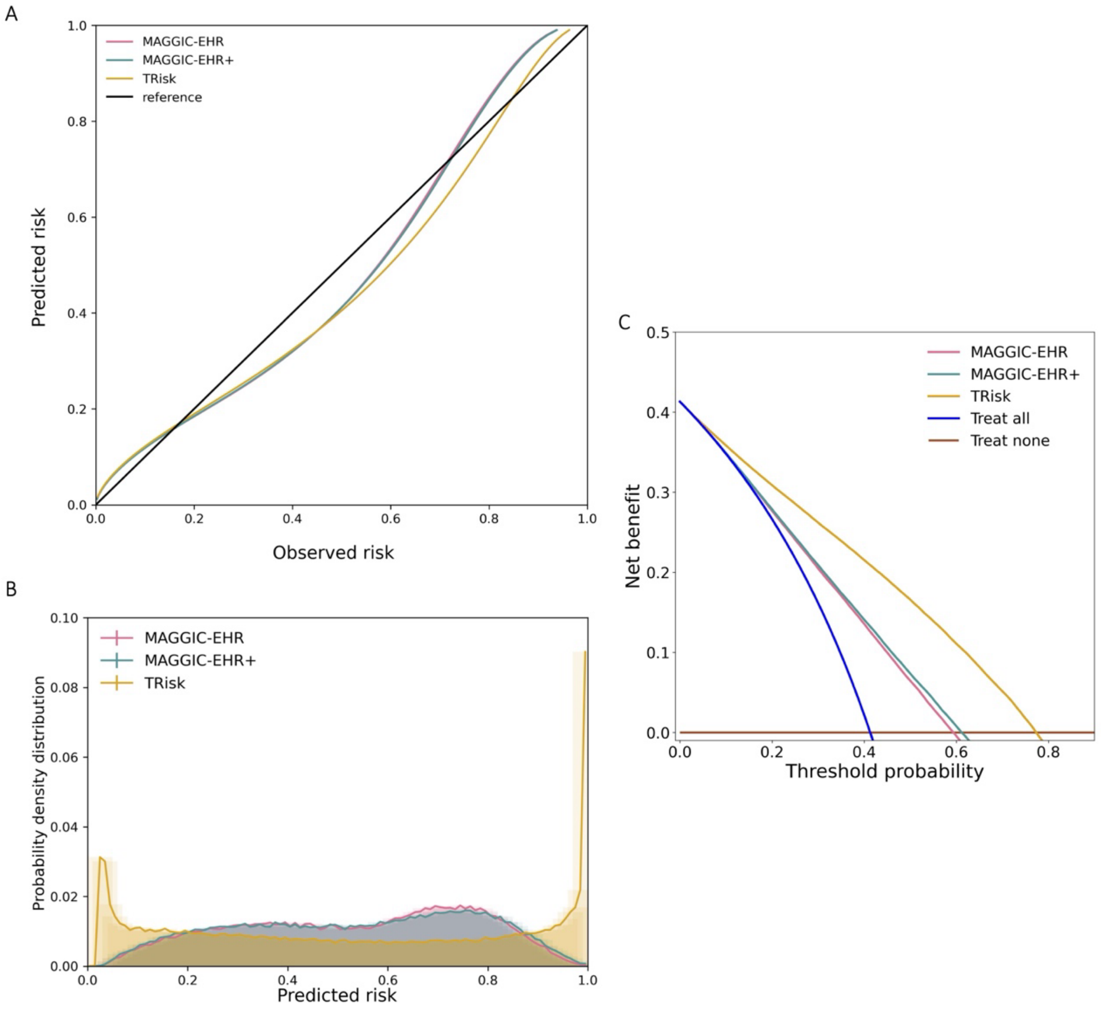

Figure S4. Calibration curves, distribution of predicted risk of models, decision curve analyses for 36-month all-cause mortality across all models on UK validation data.

(A) Calibration curves, (B) distribution of predicted risk, and (C) decision curve analyses are presented for all models. Decision curve analysis (including censored observations) has been conducted for all models. Threshold probability is shown on the x-axis and the net benefit, a function of threshold probability, is shown on the y-axis and is the difference between the proportion of true positives and false positives weighted by odds of the respective decision threshold.

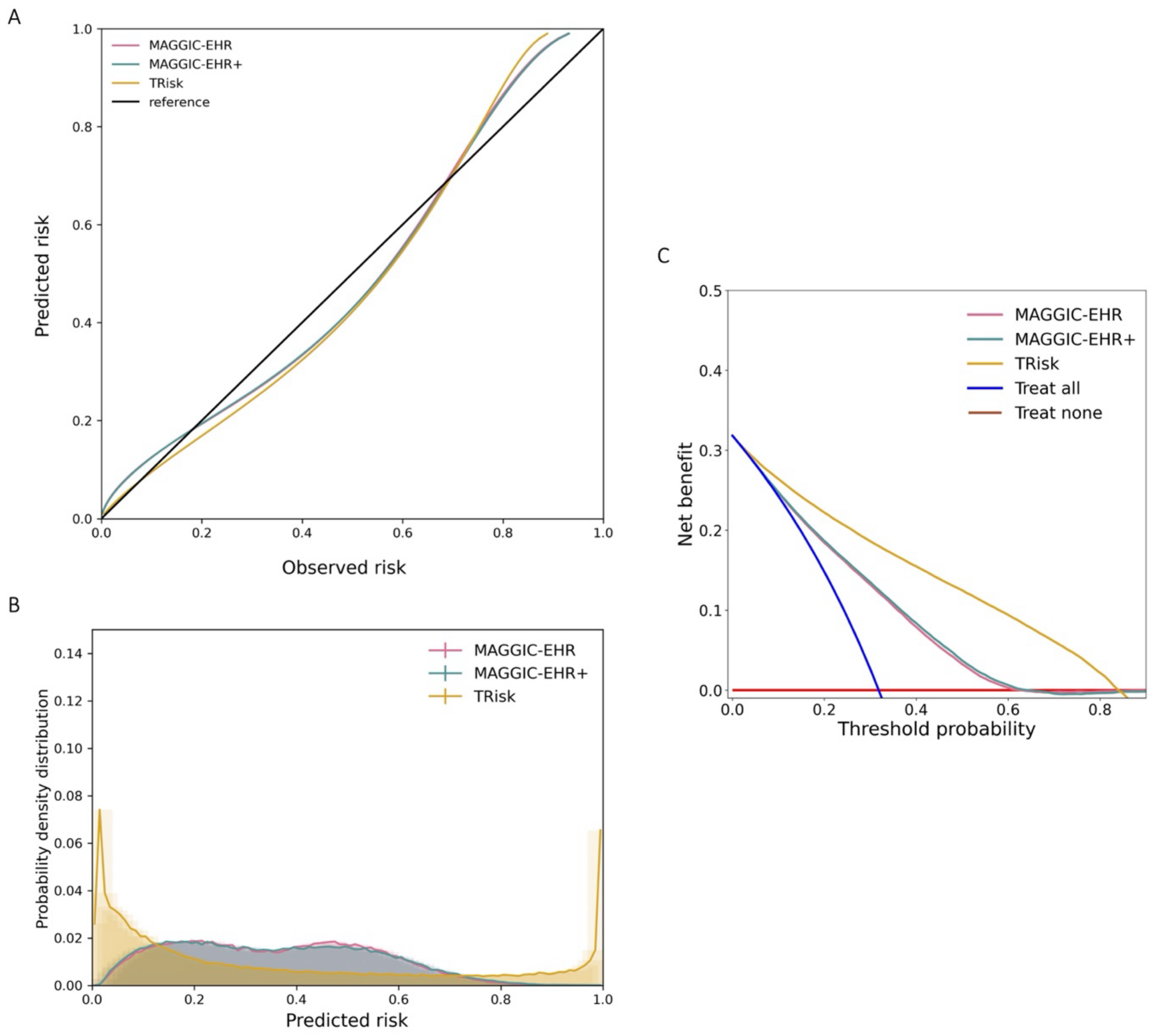

Figure S5. Calibration curves, distribution of predicted risk of models, decision curve analyses for 12-month all-cause mortality across all models on UK validation data.

(A) Calibration curves, (B) distribution of predicted risk, and (C) decision curve analyses are presented for all models. Decision curve analysis (including censored observations) has been conducted for all models. Threshold probability is shown on the x-axis and the net benefit, a function of threshold probability, is shown on the y-axis and is the difference between the proportion of true positives and false positives weighted by odds of the respective decision threshold.

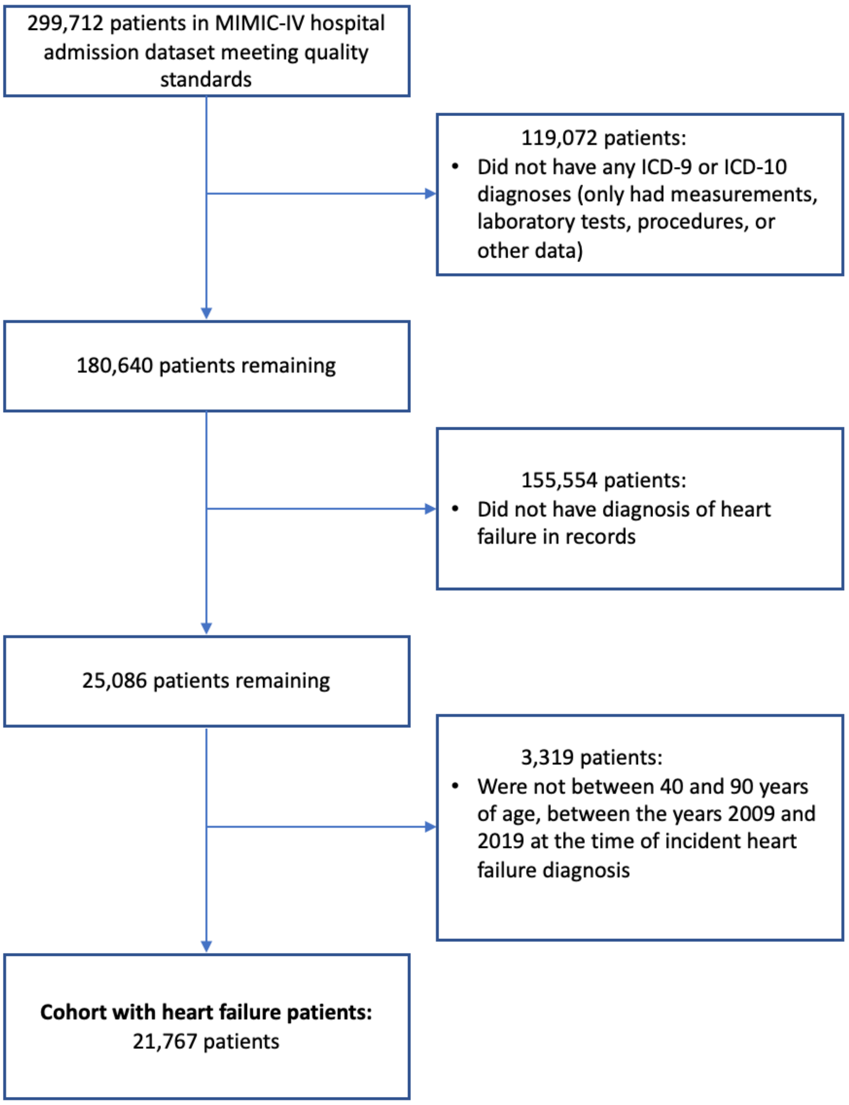

Figure S6. Cohort selection for MIMIC-IV hospital admissions validation dataset.
MIMIC-IV: Medical Information Mart for Intensive Care-IV dataset.

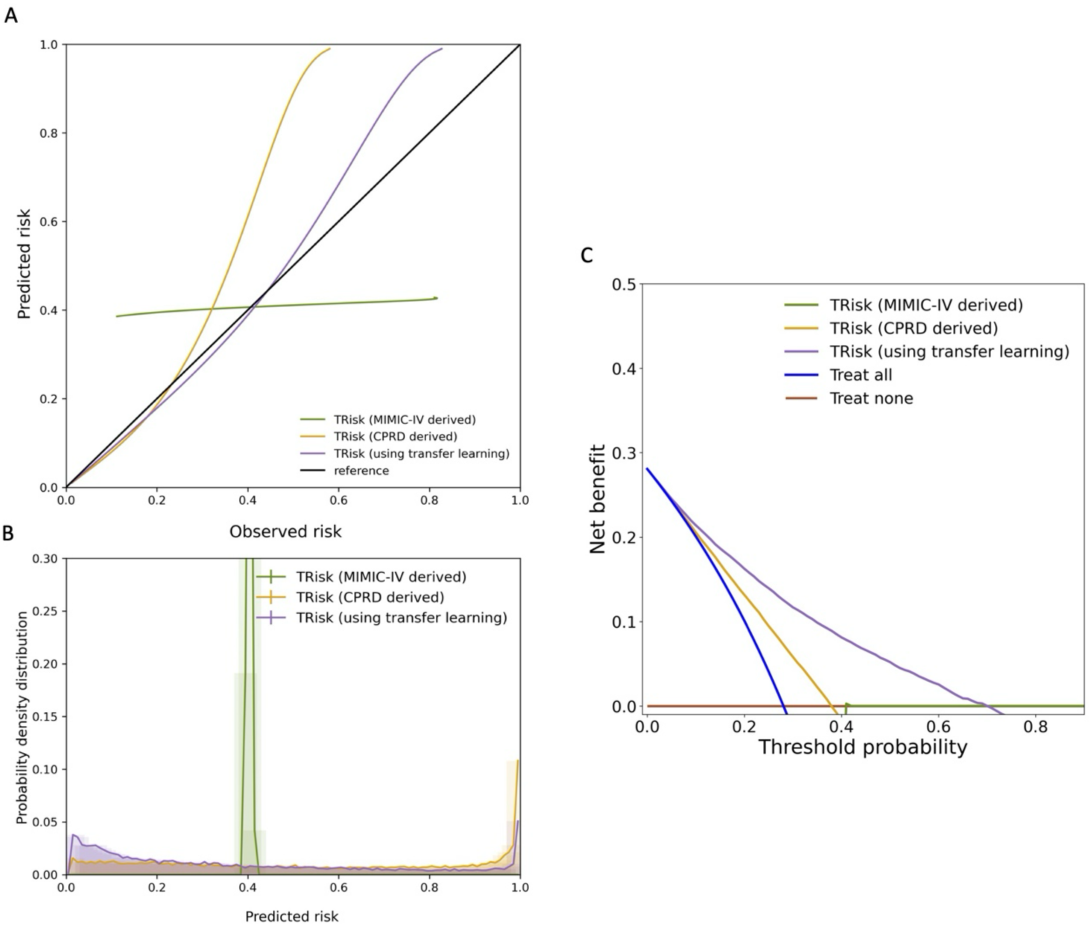

Figure S7. Calibration curves, distribution of predicted risk of models, and decision curve analysis for 12-month all-cause mortality risk prediction on USA validation data.

(A) Calibration curves, (B) distribution of predicted risk, and (C) decision curve analyses are presented for all models. Decision curve analysis (including censored observations) has been conducted for all models. Threshold probability is shown on the x-axis and the net benefit, a function of threshold probability, is shown on the y-axis and is the difference between the proportion of true positives and false positives weighted by odds of the respective decision threshold. TRisk (MIMIC-IV derived) is randomly initialised and trained on MIMIC-IV fine-tuning dataset. TRisk (CPRD derived) is trained on CPRD derivation cohort. TRisk (using transfer learning) is trained on CPRD derivation cohort and fine-tuned on MIMIC-IV fine-tuning dataset. All models are validated on MIMIC-IV validation dataset.

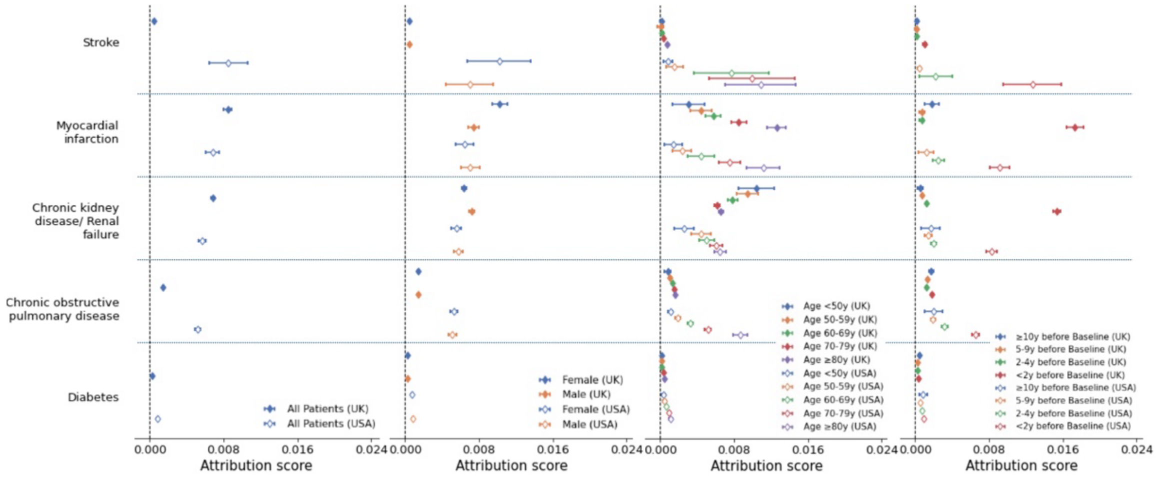

Figure S8. Average contribution for validated risk factors contributing to mortality risk prediction on UK and USA validation cohort datasets.

Point estimates of contribution values and associated 95% confidence intervals are presented for each encounter. Average contribution scores are calculated with appropriate 95% confidence interval across all patients, stratified by sex, stratified by age at first encounter, and time before baseline of first encounter.

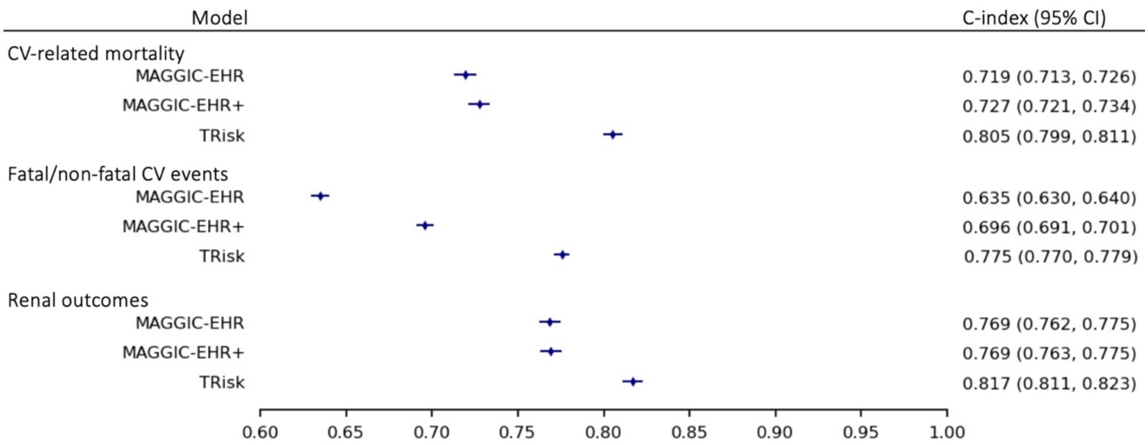

Figure S9. Discriminative performance of models for 36-month risk prediction of various outcomes on UK validation data.

Discrimination is provided in this forest plot as assessed by C-index with 95% confidence intervals (CI) for various outcome investigations at 36-month timepoint; CV: cardiovascular.

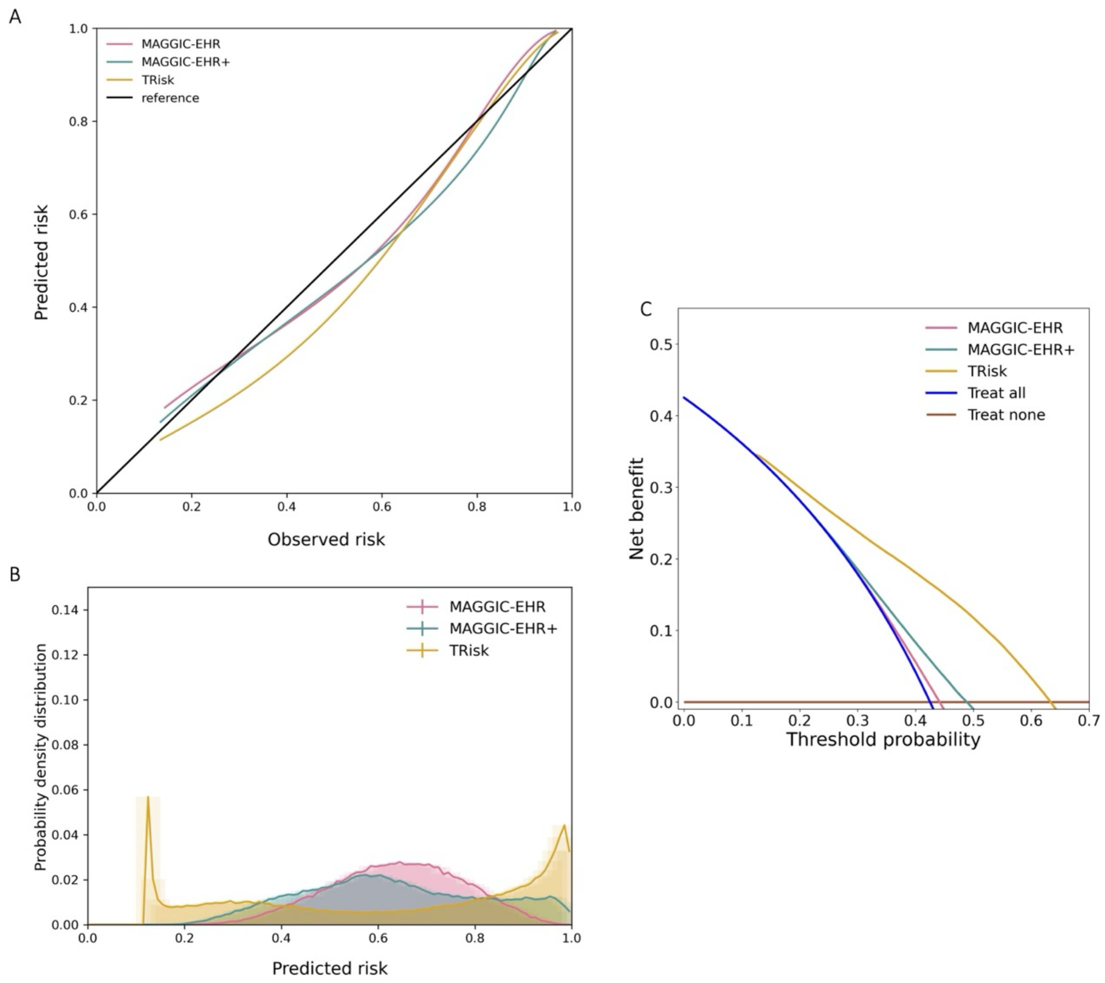

Figure S10. Calibration curves, distribution of predicted risk of models, decision curve analyses 36-month non-fatal/fatal cardiovascular event prediction on UK validation data.

(A) Calibration curves, (B) distribution of predicted risk, and (C) decision curve analyses are presented for all models. Decision curve analysis (including censored observations) has been conducted for all models. Threshold probability is shown on the x-axis and the net benefit, a function of threshold probability, is shown on the y-axis and is the difference between the proportion of true positives and false positives weighted by odds of the respective decision threshold.

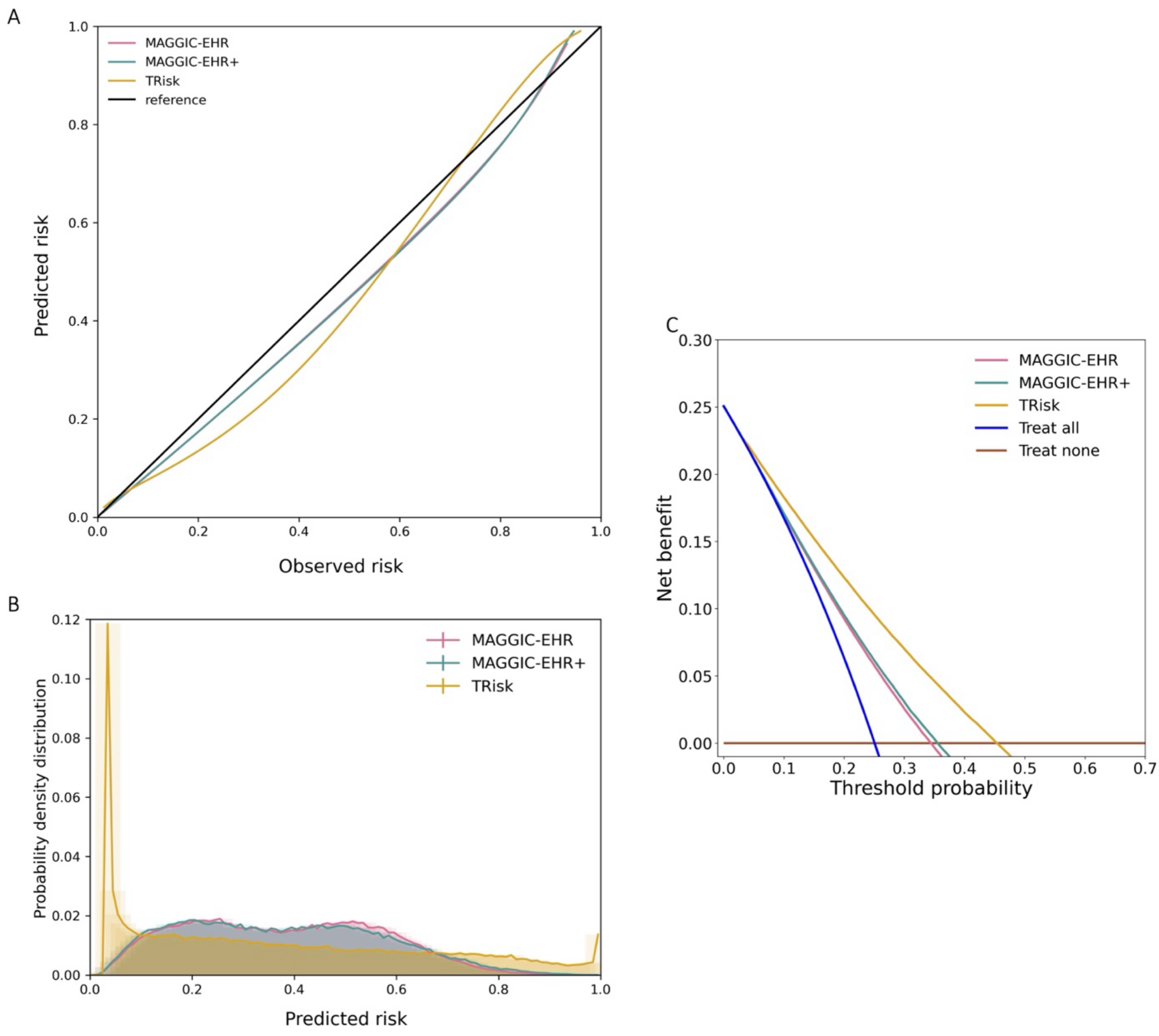

Figure S11. Calibration curves, distribution of predicted risk of models, decision curve analyses 36-month CV-related mortality prediction on UK validation data.

(A) Calibration curves, (B) distribution of predicted risk, and (C) decision curve analyses are presented for all models. Decision curve analysis (including censored observations) has been conducted for all models. Threshold probability is shown on the x-axis and the net benefit, a function of threshold probability, is shown on the y-axis and is the difference between the proportion of true positives and false positives weighted by odds of the respective decision threshold.

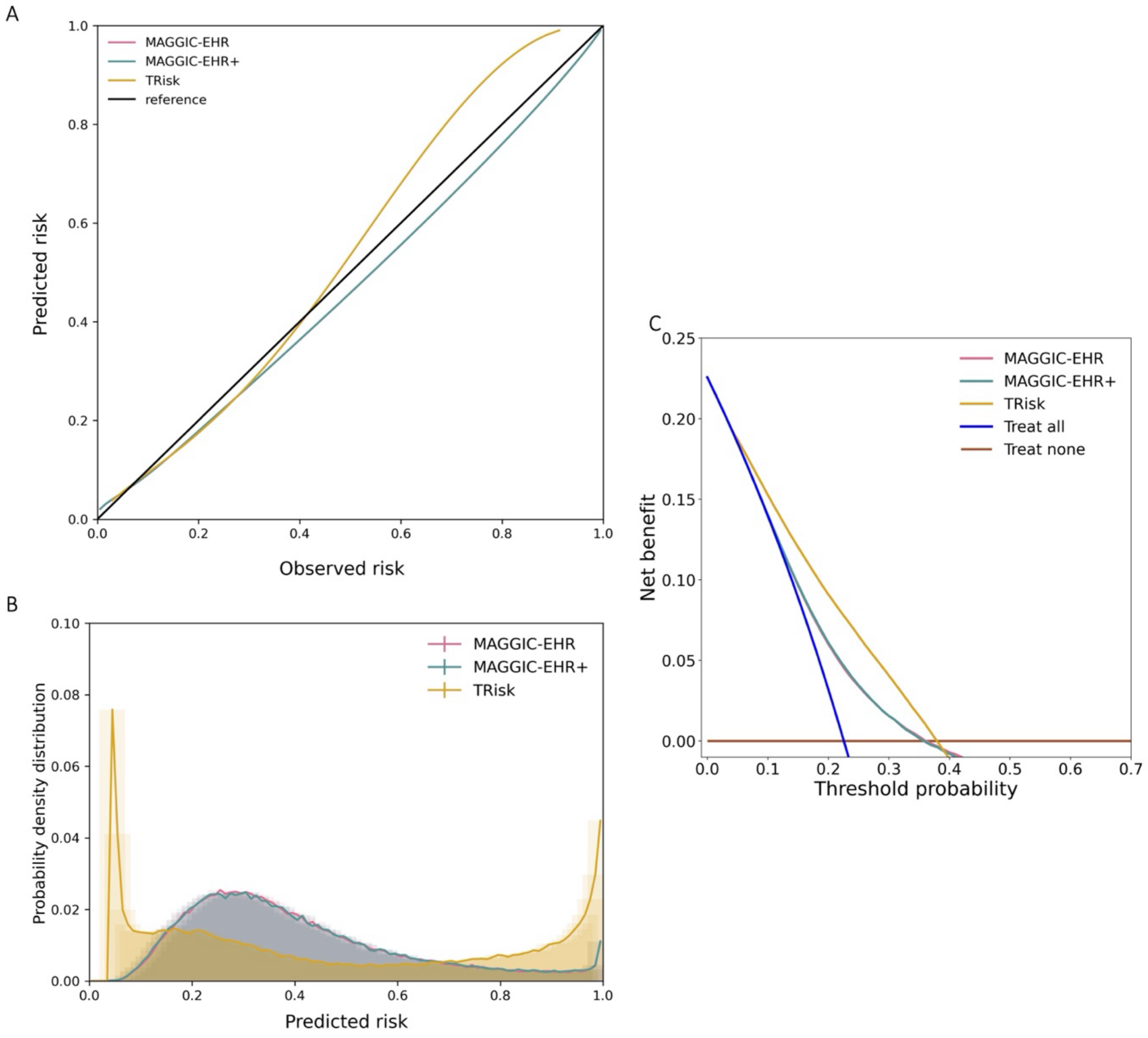

Figure S12. Calibration curves, distribution of predicted risk of models, decision curve analyses 36-month renal outcomes prediction on UK validation data.

(A) Calibration curves, (B) distribution of predicted risk, and (C) decision curve analyses are presented for all models. Decision curve analysis (including censored observations) has been conducted for all models. Threshold probability is shown on the x-axis and the net benefit, a function of threshold probability, is shown on the y-axis and is the difference between the proportion of true positives and false positives weighted by odds of the respective decision threshold.

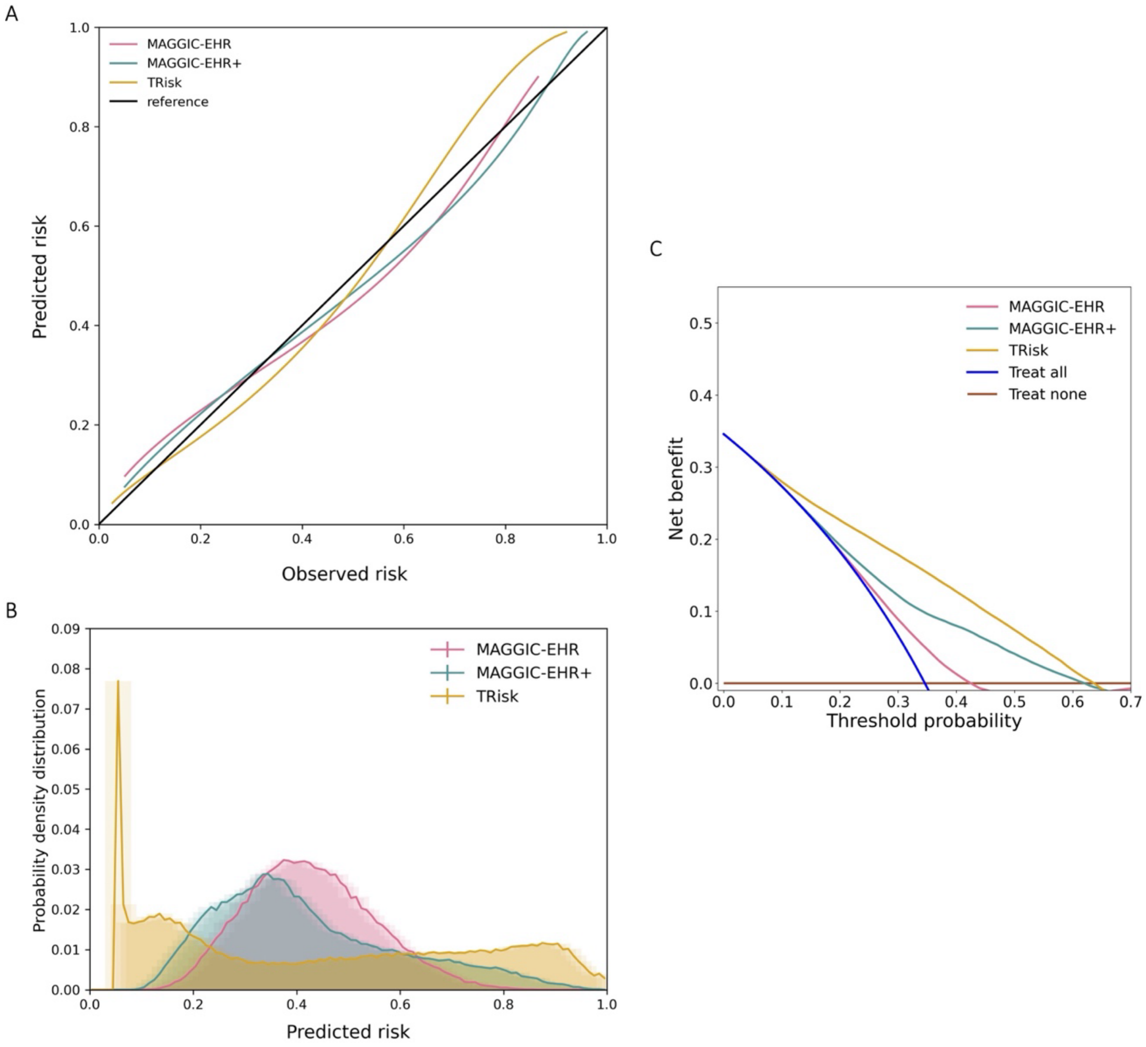

Figure S13. Calibration curves, distribution of predicted risk of models, decision curve analyses 12-month non-fatal/fatal cardiovascular event prediction on UK validation data.

(A) Calibration curves, (B) distribution of predicted risk, and (C) decision curve analyses are presented for all models. Decision curve analysis (including censored observations) has been conducted for all models. Threshold probability is shown on the x-axis and the net benefit, a function of threshold probability, is shown on the y-axis and is the difference between the proportion of true positives and false positives weighted by odds of the respective decision threshold.

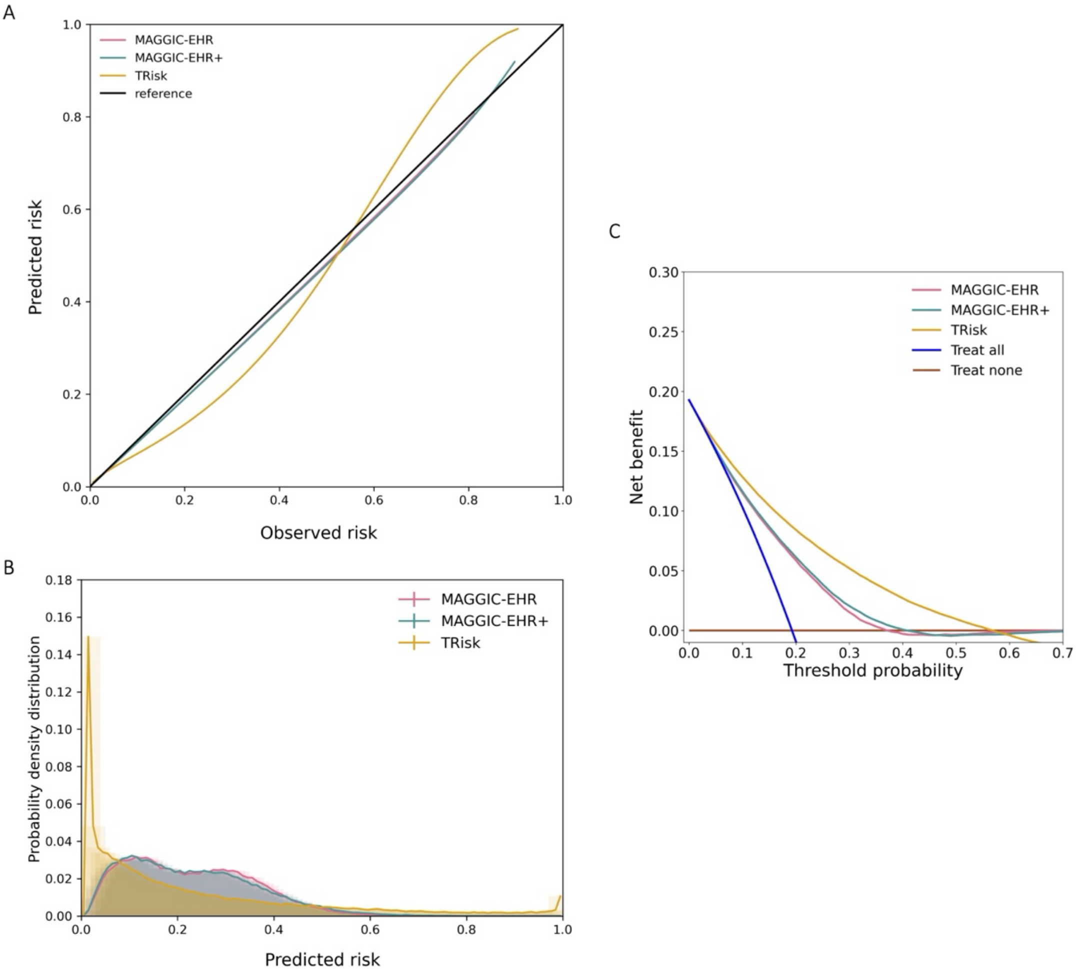

Figure S14. Calibration curves, distribution of predicted risk of models, decision curve analyses 12-month CV-related mortality prediction on UK validation data.

(A) Calibration curves, (B) distribution of predicted risk, and (C) decision curve analyses are presented for all models. Decision curve analysis (including censored observations) has been conducted for all models. Threshold probability is shown on the x-axis and the net benefit, a function of threshold probability, is shown on the y-axis and is the difference between the proportion of true positives and false positives weighted by odds of the respective decision threshold.

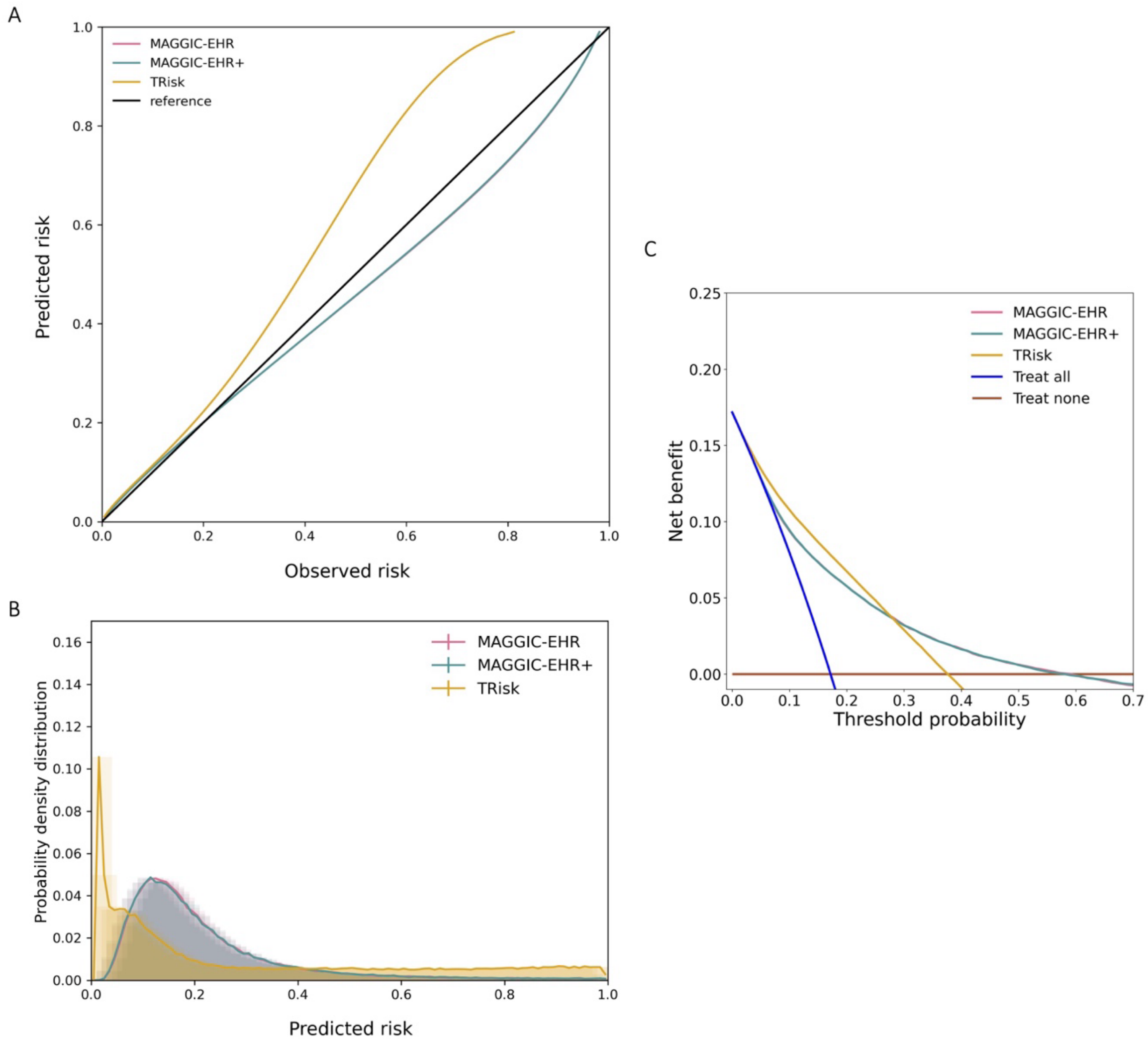

Figure S15. Calibration curves, distribution of predicted risk of models, decision curve analyses 12-month renal outcomes prediction on UK validation data.

(A) Calibration curves, (B) distribution of predicted risk, and (C) decision curve analyses are presented for all models. Decision curve analysis (including censored observations) has been conducted for all models. Threshold probability is shown on the x-axis and the net benefit, a function of threshold probability, is shown on the y-axis and is the difference between the proportion of true positives and false positives weighted by odds of the respective decision threshold.

Supplementary Tables

Table S1. Model hyperparameters and other settings for TRisk

**Hyperparameters**	**Value**
Number of layers	6
Maximum sequence length	512
Hidden size	150
Hidden dropout rate	0.3
Attention dropout rate	0.4
Number of attention heads	6
Intermediate size	108
Pooling layer size	150
Hidden activation function	Gaussian Error Linear Unit (GELU) function
Learning rate	0.00008
Weight decay	0.02
Warmup proportion (Adam optimiser hyperparameter)	0.1
Embedding concatenation	True
Embedding non-linear transformation	Hyperbolic tangent function
λ weightage for explicit calibration-related modelling	2.0
Interpolation for explicit calibration-related modelling	True
Time bins for explicit calibration-related modelling	48 (i.e., 1 per month)

Table S2. Area under the precision-recall curve (AUPRC) metrics for all models for all 36-month risk prediction investigations on UK validation data.

		AUPRC
All-cause mortality	MAGGIC-EHR	0.623
	MAGGIC-EHR+	0.633
	TRisk	0.797
	Model that presents chance-level performance	0.411
Non-fatal/fatal cardiovascular events	MAGGIC-EHR	0.510
	MAGGIC-EHR+	0.616
	TRisk	0.700
	Model that presents chance-level performance	0.427
Cardiovascular-related mortality	MAGGIC-EHR	0.388
	MAGGIC-EHR+	0.403
	TRisk	0.524
	Model that presents chance-level performance	0.250
Renal outcomes	MAGGIC-EHR	0.478
	MAGGIC-EHR+	0.478
	TRisk	0.529
	Model that presents chance-level performance	0.224

AUPRC: area under precision-recall curve

Table S3. Subgroup discrimination analysis for 36-month all-cause mortality prediction investigations on UK validation data

Analysis	Model	Subgroup	Count	Concordance index (95% CI)	AUPRC
Sex	MAGGIC-EHR	Male	53,938	0.741 (0.734, 0.748)	0.634
	MAGGIC-EHR	Female	45,444	0.712 (0.705, 0.719)	0.609
	MAGGIC-EHR+	Male	53,938	0.747 (0.741, 0.754)	0.643
	MAGGIC-EHR+	Female	45,444	0.719 (0.711, 0.726)	0.619
	TRisk	Male	53,938	0.849 (0.843, 0.854)	0.801
	TRisk	Female	45,444	0.841 (0.835, 0.847)	0.794
Age	MAGGIC-EHR	<60 years	15,738	0.724 (0.710, 0.737)	0.570
	MAGGIC-EHR	≥60 years	83,644	0.729 (0.724, 0.734)	0.633
	MAGGIC-EHR+	<60 years	15,738	0.733 (0.719, 0.746)	0.580
	MAGGIC-EHR+	≥60 years	83,644	0.735 (0.730, 0.740)	0.642
	TRisk	<60 years	15,738	0.845 (0.834, 0.856)	0.745
	TRisk	≥60 years	83,644	0.844 (0.840, 0.848)	0.804
Baseline systolic blood pressure	MAGGIC-EHR	<130 mmHg	37,798	0.744 (0.736, 0.752)	0.632
	MAGGIC-EHR	≥130 mmHg	61,584	0.718 (0.712, 0.724)	0.617
	MAGGIC-EHR+	<130 mmHg	37,798	0.752 (0.744, 0.760)	0.644
	MAGGIC-EHR+	≥130 mmHg	61,584	0.724 (0.717, 0.730)	0.625
	TRisk	<130 mmHg	37,798	0.848 (0.842, 0.855)	0.795
	TRisk	≥130 mmHg	61,584	0.843 (0.838, 0.848)	0.799
HF subtype	MAGGIC-EHR	Preserved	4,350	0.662 (0.623, 0.700)	0.271
	MAGGIC-EHR	Reduced	21,468	0.681 (0.667, 0.695)	0.366
	MAGGIC-EHR+	Preserved	4,350	0.674 (0.636, 0.712)	0.285
	MAGGIC-EHR+	Reduced	21,468	0.690 (0.676, 0.704)	0.378
	TRisk	Preserved	4,350	0.818 (0.787, 0.850)	0.511
	TRisk	Reduced	21,468	0.799 (0.786, 0.811)	0.575
Baseline disease	MAGGIC-EHR	Diabetes	29,659	0.709 (0.700, 0.718)	0.607
	MAGGIC-EHR+	Diabetes	29,659	0.715 (0.706, 0.723)	0.613
	TRisk	Diabetes	29,659	0.829 (0.822, 0.837)	0.785
	MAGGIC-EHR	Atrial fibrillation	48,033	0.713 (0.706, 0.720)	0.629
	MAGGIC-EHR+	Atrial fibrillation	48,033	0.718 (0.711, 0.725)	0.636
	TRisk	Atrial fibrillation	48,033	0.833 (0.827, 0.838)	0.797
	MAGGIC-EHR	Myocardial infarction	28,500	0.729 (0.719, 0.738)	0.599
	MAGGIC-EHR+	Myocardial infarction	28,500	0.738 (0.728, 0.747)	0.611
	TRisk	Myocardial infarction	28,500	0.830 (0.822, 0.838)	0.760
Baseline medication	MAGGIC-EHR	Beta-blockers	34,258	0.715 (0.707, 0.724)	0.615
	MAGGIC-EHR+	Beta-blockers	34,258	0.721 (0.712, 0.729)	0.623
	TRisk	Beta-blockers	34,258	0.828 (0.821, 0.835)	0.782
	MAGGIC-EHR	Angiotensin-converting-enzyme inhibitors	52,799	0.726 (0.718, 0.733)	0.568
	MAGGIC-EHR+	Angiotensin-converting-enzyme inhibitors	52,799	0.734 (0.727, 0.741)	0.584
	TRisk	Angiotensin-converting-enzyme inhibitors	52,799	0.834 (0.828, 0.840)	0.751
	MAGGIC-EHR	Angiotensin receptor blockers	26,205	0.720 (0.709, 0.730)	0.559
	MAGGIC-EHR+	Angiotensin receptor blockers	26,205	0.726 (0.716, 0.736)	0.570
	TRisk	Angiotensin receptor blockers	26,205	0.837 (0.829, 0.846)	0.751

HF: heart failure; AUPRC: area under precision-recall curve; CI: confidence interval

Table S4. Integrated calibration index (ICI) for various 36-month risk prediction investigations across all models on UK validation data

Integrated Calibration Index (ICI)*				
	All-cause mortality	Non-fatal/fatal cardiovascular events	Cardiovascular-related mortality	Renal outcomes
MAGGIC-EHR	0.0478	0.0433	0.0102	0.0381
MAGGIC-EHR+	0.0496	0.0543	0.0110	0.0380
TRisk	0.0419	0.0503	0.0501	0.0481

**lower is better*

Table S5. Ablation analyses on TRisk model: prognostication of 36-month all-cause mortality in UK dataset

	Discrimination: Concordance index (95% CI)	Discrimination: AUPRC	Calibration: ICI*
TRisk (for reference)	0.845 (0.841, 0.849)	0.797	0.042
TRisk without Explicit Calibration [XCal]	0.843 (0.838, 0.847)	0.790	0.056
TRisk without Concatenated Embeddings	0.828 (0.824, 0.833)	0.776	0.043
TRisk without Explicit Calibration [XCal] And Concatenated Embeddings	0.828 (0.823, 0.832)	0.777	0.054

**lower is better; AUPRC: area under the precision-recall curve; 95% CI: 95% confidence intervals*

Table S6. Random survival forest modelling hyperparameter search

Number of estimators	Minimum number of samples that must be in a leaf node	Minimum number of samples required in a node before it is allowed to split	Number of features considered when looking for the best split at each node	C-index point-estimate
100	50	100	sqrt	0.752617246
100	50	100	0.1	0.753864594
100	50	200	sqrt	0.752370671
100	50	200	0.1	0.753261971
100	100	100	sqrt	0.746202714
100	100	100	0.1	0.746892766
100	100	200	sqrt	0.746202714
100	100	200	0.1	0.746892766
100	200	100	sqrt	0.738594258
100	200	100	0.1	0.737901862
100	200	200	sqrt	0.738594258
100	200	200	0.1	0.737901862
200	50	100	sqrt	0.753742437
200	50	100	0.1	0.754493202
200	50	200	sqrt	0.753938357
200	50	200	0.1	0.753735021
200	100	100	sqrt	0.748324291
200	100	100	0.1	0.747337044
200	100	200	sqrt	0.748324291
200	100	200	0.1	0.747337044
200	200	100	sqrt	0.740522207
200	200	100	0.1	0.738560806
200	200	200	sqrt	0.740522207
200	200	200	0.1	0.738560806
300	50	100	sqrt	0.75405675
**300**	**50**	**100**	**0.1**	**0.754936175**
300	50	200	sqrt	0.754070338
300	50	200	0.1	0.753889221
300	100	100	sqrt	0.748457862
300	100	100	0.1	0.747390898
300	100	200	sqrt	0.748457862
300	100	200	0.1	0.747390898
300	200	100	sqrt	0.741098907
300	200	100	0.1	0.738523057
300	200	200	sqrt	0.741098907
300	200	200	0.1	0.738523057
Bolded model is best				

Table S7. Random survival forest modelling: prognostication of 36-month all-cause mortality in UK dataset

	Discrimination: Concordance index (95% CI)	Discrimination: AUPRC	Calibration: ICI*
RSF	0.754 (0.749; 0.759)	0.670	0.076

**lower is better; AUPRC: area under the precision-recall curve; 95% CI: 95% confidence intervals*

Table S8. Integrated calibration index (ICI) for various outcome 12-month risk prediction investigations across all models on UK validation data

Integrated Calibration Index (ICI)*				
	All-cause mortality	Non-fatal/fatal cardiovascular events	Cardiovascular-related mortality	Renal outcomes
MAGGIC-EHR	0.0527	0.0432	0.0102	0.0130
MAGGIC-EHR+	0.0510	0.0254	0.0110	0.0128
TRisk	0.0386	0.0387	0.0431	0.0598

**lower is better*

Table S9. Impact analyses at the various decision thresholds for 12- and 36-month all-cause mortality prediction on UK validation data

Threshold	Timepoint	Models	Metrics				
			PP	FP	FN	PPV	Sensitivity
**0.25**	12-month	MAGGIC-EHR	64667	36,708	3,675	0.432	0.884
		TRisk	46,810	19,894	4,718	0.575	0.851
	36-month	MAGGIC-EHR	84,882	4,5714	1,889	0.461	0.954
		TRisk	70,895	31,873	2,035	0.55	0.95
**0.50**	12-month	MAGGIC-EHR	22,924	9,882	18,592	0.569	0.412
		TRisk	30,769	9,183	10,048	0.702	0.682
	36-month	MAGGIC-EHR	56,158	24,871	9,770	0.557	0.762
		TRisk	50,848	17,178	7,387	0.662	0.821
**0.75**	12-month	MAGGIC-EHR	676	213	31,171	0.685	0.015
		TRisk	19,984	3,912	15,562	0.804	0.508
	36-month	MAGGIC-EHR	20,237	6,717	27,537	0.668	0.329
		TRisk	33,412	7,920	15,565	0.763	0.621

PP: predicted positive; FP: false positives; FN: false negatives; PPV: positive predictive value

Table S10. Population characteristics of the MIMIC-IV cohort

	Fine-tuning: 8,707 patients (40%)	Validation: 13,060 patients (60%)
Women (%)	4,789 (55.0)	7,211 (55.2)
Median age in years (IQI)	74 (65, 83)	74 (65, 82)
Smoking status		
Current/ex-smoker (%) [Ω]	3,891 (44.7)	5,853 (44.8)
Non-smoker (%)	4,816 (55.3)	7,207 (55.2)
Median creatinine (µmol/L) (IQI) [†]	88.4 (79.6, 106.1)	88.4 (79.6, 106.1)
Median sodium (mmol/L) (IQI) [†]	138.8 (136.8, 140.7)	138.8 (136.7, 140.7)
Median BMI (kg/m^2) (IQI) [†]	28.3 (24.4, 33.2)	28.4 (24.3, 33.5)
Median SBP (mmHg) (IQI) [†]	129.0 (119.2, 139.7)	128.9 (119.1, 139.3)
<18 months after HF (%)	7,158 (82.2)	10,749 (82.3)
Diseases at baseline		
Diabetes	3,369 (38.7)	4,984 (38.2)
COPD	2,280 (26.2)	3,323 (25.4)
Atrial fibrillation	4,124 (47.4)	6,131 (46.9)
Stroke	568 (6.5)	877 (6.7)
Medication use at baseline		
Beta blockers	6,389 (73.4)	9,678 (74.1)

%: percent; IQI: interquartile interval; BMI: body mass index; SBP: systolic blood pressure; COPD: Chronic obstructive pulmonary disease; AF: atrial fibrillation; [Ω] current/ex-smoker status identified by F17 and Z87.891 ICD-10 codes; [†]indicates missing variables; BMI (41.7% missingness), SBP (44.1%), creatinine (4.5%), sodium (4.7%).

Table S11. Area under the precision-recall curve (AUPRC) metrics for all models for all 12- and 36-month risk prediction of all-cause mortality prediction on MIMIC-IV validation data

AUPRC		
Model	12-month	36-month
TRisk (MIMIC-IV derived)	0.403	0.361
TRisk (CPRD derived)	0.541	0.551
TRisk (using transfer learning)	0.690	0.693
Model that presents chance-level performance (for reference)	0.277	0.340

Table S12. Integrated calibration index (ICI) for 12- and 36-month risk prediction of all-cause mortality on MIMIC-IV validation data across all models

**Integrated calibration index (ICI)***		
Model	12-month	36-month
TRisk (MIMIC-IV derived)	0.088	0.068
TRisk (CPRD derived)	0.312	0.166
TRisk (using transfer learning)	0.051	0.053

**lower is better*

Table S13. Subgroup discrimination analysis for 36-month cardiovascular-related mortality prediction investigations on UK validation data

Analysis	Model	Subgroup	Count	Concordance index (95% CI)	AUPRC
Sex	MAGGIC-EHR	Male	53,938	0.729 (0.720, 0.737)	0.403
	MAGGIC-EHR	Female	45,444	0.708 (0.698, 0.717)	0.366
	MAGGIC-EHR+	Male	53,938	0.735 (0.726, 0.743)	0.416
	MAGGIC-EHR+	Female	45,444	0.718 (0.709, 0.728)	0.386
	TRisk	Male	53,938	0.810 (0.802, 0.817)	0.544
	TRisk	Female	45,444	0.800 (0.792, 0.809)	0.518
Age	MAGGIC-EHR	<60 years	15,738	0.725 (0.707, 0.743)	0.352
	MAGGIC-EHR	≥60 years	83,644	0.719 (0.712, 0.725)	0.396
	MAGGIC-EHR+	<60 years	15,738	0.734 (0.716, 0.752)	0.364
	MAGGIC-EHR+	≥60 years	83,644	0.726 (0.720, 0.733)	0.411
	TRisk	<60 years	15,738	0.802 (0.786, 0.818)	0.471
	TRisk	≥60 years	83,644	0.804 (0.798, 0.810)	0.540
Baseline systolic blood pressure	MAGGIC-EHR	<130 mmHg	37,798	0.734 (0.724, 0.744)	0.384
	MAGGIC-EHR	≥130 mmHg	61,584	0.710 (0.702, 0.718)	0.390
	MAGGIC-EHR+	<130 mmHg	37,798	0.742 (0.732, 0.753)	0.401
	MAGGIC-EHR+	≥130 mmHg	61,584	0.718 (0.710, 0.726)	0.404
	TRisk	<130 mmHg	37,798	0.806 (0.797, 0.816)	0.509
	TRisk	≥130 mmHg	61,584	0.804 (0.797, 0.811)	0.544
HF subtype	MAGGIC-EHR	Preserved	4,350	0.665 (0.613, 0.717)	0.156
	MAGGIC-EHR	Reduced	21,468	0.688 (0.671, 0.705)	0.261
	MAGGIC-EHR+	Preserved	4,350	0.687 (0.636, 0.738)	0.171
	MAGGIC-EHR+	Reduced	21,468	0.698 (0.681, 0.715)	0.270
	TRisk	Preserved	4,350	0.806 (0.762, 0.850)	0.289
	TRisk	Reduced	21,468	0.777 (0.761, 0.792)	0.392
Baseline disease	MAGGIC-EHR	Diabetes	29,659	0.702 (0.691, 0.713)	0.406
	MAGGIC-EHR+	Diabetes	29,659	0.706 (0.695, 0.717)	0.415
	TRisk	Diabetes	29,659	0.789 (0.779, 0.799)	0.550
	MAGGIC-EHR	Atrial fibrillation	48,033	0.701 (0.692, 0.709)	0.404
	MAGGIC-EHR+	Atrial fibrillation	48,033	0.708 (0.700, 0.717)	0.417
	TRisk	Atrial fibrillation	48,033	0.789 (0.781, 0.797)	0.539
	MAGGIC-EHR	Myocardial infarction	28,500	0.724 (0.713, 0.735)	0.443
	MAGGIC-EHR+	Myocardial infarction	28,500	0.730 (0.720, 0.741)	0.446
	TRisk	Myocardial infarction	28,500	0.797 (0.787, 0.807)	0.551
Baseline medication	MAGGIC-EHR	Beta-blockers	34,258	0.712 (0.701, 0.722)	0.417
	MAGGIC-EHR+	Beta-blockers	34,258	0.719 (0.709, 0.729)	0.431
	TRisk	Beta-blockers	34,258	0.791 (0.782, 0.800)	0.544
	MAGGIC-EHR	Angiotensin-converting-enzyme inhibitors	52,799	0.723 (0.714, 0.732)	0.375
	MAGGIC-EHR+	Angiotensin-converting-enzyme inhibitors	52,799	0.732 (0.723, 0.741)	0.392
	TRisk	Angiotensin-converting-enzyme inhibitors	52,799	0.800 (0.792, 0.808)	0.510
	MAGGIC-EHR	Angiotensin receptor blockers	26,205	0.717 (0.705, 0.730)	0.374
	MAGGIC-EHR+	Angiotensin receptor blockers	26,205	0.727 (0.715, 0.740)	0.392
	TRisk	Angiotensin receptor blockers	26,205	0.803 (0.792, 0.814)	0.513

HF: heart failure; AUPRC: area under precision-recall curve; CI: confidence interval

Table S14. Subgroup discrimination analysis for 36-month non-fatal/fatal cardiovascular event prediction investigations on UK validation data

Analysis	Model	Subgroup	Count	Concordance index (95% CI)	AUPRC
Sex	MAGGIC-EHR	Male	53,938	0.631 (0.624, 0.638)	0.539
	MAGGIC-EHR	Female	45,444	0.630 (0.623, 0.639)	0.448
	MAGGIC-EHR+	Male	53,938	0.686 (0.680, 0.693)	0.641
	MAGGIC-EHR+	Female	45,444	0.702 (0.694, 0.710)	0.572
	TRisk	Male	53,938	0.763 (0.757, 0.769)	0.715
	TRisk	Female	45,444	0.786 (0.779, 0.793)	0.677
Age	MAGGIC-EHR	<60 years	15,738	0.631 (0.617, 0.645)	0.471
	MAGGIC-EHR	≥60 years	83,644	0.633 (0.627, 0.638)	0.515
	MAGGIC-EHR+	<60 years	15,738	0.712 (0.699, 0.726)	0.592
	MAGGIC-EHR+	≥60 years	83,644	0.691 (0.686, 0.697)	0.619
	TRisk	<60 years	15,738	0.789 (0.777, 0.801)	0.679
	TRisk	≥60 years	83,644	0.771 (0.766, 0.776)	0.703
Baseline systolic blood pressure	MAGGIC-EHR	<130 mmHg	37,798	0.640 (0.632, 0.649)	0.515
	MAGGIC-EHR	≥130 mmHg	61,584	0.632 (0.625, 0.638)	0.507
	MAGGIC-EHR+	<130 mmHg	37,798	0.700 (0.692, 0.708)	0.617
	MAGGIC-EHR+	≥130 mmHg	61,584	0.694 (0.687, 0.700)	0.615
	TRisk	<130 mmHg	37,798	0.772 (0.764, 0.779)	0.689
	TRisk	≥130 mmHg	61,584	0.777 (0.771, 0.782)	0.706
HF subtype	MAGGIC-EHR	Preserved	4,350	0.634 (0.605, 0.664)	0.424
	MAGGIC-EHR	Reduced	21,468	0.622 (0.611, 0.634)	0.534
	MAGGIC-EHR+	Preserved	4,350	0.708 (0.681, 0.736)	0.532
	MAGGIC-EHR+	Reduced	21,468	0.693 (0.683, 0.704)	0.625
	TRisk	Preserved	4,350	0.792 (0.767, 0.817)	0.655
	TRisk	Reduced	21,468	0.747 (0.737, 0.757)	0.707
Baseline disease	MAGGIC-EHR	Diabetes	29,659	0.620 (0.611, 0.629)	0.554
	MAGGIC-EHR+	Diabetes	29,659	0.665 (0.657, 0.674)	0.640
	TRisk	Diabetes	29,659	0.754 (0.746, 0.762)	0.731
	MAGGIC-EHR	Atrial fibrillation	48,033	0.630 (0.622, 0.637)	0.512
	MAGGIC-EHR+	Atrial fibrillation	48,033	0.691 (0.684, 0.698)	0.612
	TRisk	Atrial fibrillation	48,033	0.770 (0.763, 0.776)	0.695
	MAGGIC-EHR	Myocardial infarction	28,500	0.597 (0.589, 0.605)	0.700
	MAGGIC-EHR+	Myocardial infarction	28,500	0.603 (0.595, 0.611)	0.704
	TRisk	Myocardial infarction	28,500	0.682 (0.674, 0.689)	0.766
Baseline medication	MAGGIC-EHR	Beta-blockers	34,258	0.629 (0.621, 0.637)	0.568
	MAGGIC-EHR+	Beta-blockers	34,258	0.683 (0.675, 0.691)	0.672
	TRisk	Beta-blockers	34,258	0.756 (0.749, 0.764)	0.736
	MAGGIC-EHR	Angiotensin-converting-enzyme inhibitors	52,799	0.633 (0.626, 0.640)	0.539
	MAGGIC-EHR+	Angiotensin-converting-enzyme inhibitors	52,799	0.694 (0.687, 0.700)	0.643
	TRisk	Angiotensin-converting-enzyme inhibitors	52,799	0.764 (0.758, 0.770)	0.722
	MAGGIC-EHR	Angiotensin receptor blockers	26,205	0.632 (0.623, 0.642)	0.528
	MAGGIC-EHR+	Angiotensin receptor blockers	26,205	0.696 (0.687, 0.706)	0.638
	TRisk	Angiotensin receptor blockers	26,205	0.767 (0.758, 0.776)	0.719

HF: heart failure; AUPRC: area under precision-recall curve; CI: confidence interval

Table S15. Subgroup discrimination analysis for 36-month renal outcomes prediction investigations on UK validation data

Analysis	Model	Subgroup	Count	Concordance index (95% CI)	AUPRC
Sex	MAGGIC-EHR	Male	53,938	0.782 (0.774, 0.791)	0.496
	MAGGIC-EHR	Female	45,444	0.751 (0.741, 0.76)	0.455
	MAGGIC-EHR+	Male	53,938	0.783 (0.774, 0.791)	0.497
	MAGGIC-EHR+	Female	45,444	0.751 (0.742, 0.761)	0.454
	TRisk	Male	53,938	0.829 (0.821, 0.836)	0.545
	TRisk	Female	45,444	0.802 (0.793, 0.811)	0.504
Age	MAGGIC-EHR	<60 years	15,738	0.787 (0.770, 0.804)	0.480
	MAGGIC-EHR	≥60 years	83,644	0.765 (0.758, 0.771)	0.477
	MAGGIC-EHR+	<60 years	15,738	0.788 (0.772, 0.805)	0.480
	MAGGIC-EHR+	≥60 years	83,644	0.765 (0.758, 0.772)	0.477
	TRisk	<60 years	15,738	0.851 (0.836, 0.865)	0.556
	TRisk	≥60 years	83,644	0.809 (0.803, 0.816)	0.522
Baseline systolic blood pressure	MAGGIC-EHR	<130 mmHg	37,798	0.775 (0.764, 0.785)	0.455
	MAGGIC-EHR	≥130 mmHg	61,584	0.765 (0.757, 0.772)	0.489
	MAGGIC-EHR+	<130 mmHg	37,798	0.775 (0.765, 0.786)	0.456
	MAGGIC-EHR+	≥130 mmHg	61,584	0.765 (0.757, 0.773)	0.489
	TRisk	<130 mmHg	37,798	0.820 (0.810, 0.830)	0.503
	TRisk	≥130 mmHg	61,584	0.815 (0.807, 0.822)	0.538
HF subtype	MAGGIC-EHR	Preserved	4,350	0.780 (0.751, 0.809)	0.530
	MAGGIC-EHR	Reduced	21,468	0.777 (0.764, 0.790)	0.535
	MAGGIC-EHR+	Preserved	4,350	0.781 (0.752, 0.810)	0.533
	MAGGIC-EHR+	Reduced	21,468	0.776 (0.763, 0.789)	0.536
	TRisk	Preserved	4,350	0.818 (0.791, 0.845)	0.590
	TRisk	Reduced	21,468	0.807 (0.795, 0.820)	0.573
Baseline disease	MAGGIC-EHR	Diabetes	29,659	0.743 (0.733, 0.753)	0.546
	MAGGIC-EHR+	Diabetes	29,659	0.743 (0.732, 0.753)	0.546
	TRisk	Diabetes	29,659	0.803 (0.794, 0.812)	0.598
	MAGGIC-EHR	Atrial fibrillation	48,033	0.751 (0.742, 0.760)	0.486
	MAGGIC-EHR+	Atrial fibrillation	48,033	0.751 (0.742, 0.760)	0.486
	TRisk	Atrial fibrillation	48,033	0.800 (0.792, 0.808)	0.524
	MAGGIC-EHR	Myocardial infarction	28,500	0.766 (0.755, 0.777)	0.522
	MAGGIC-EHR+	Myocardial infarction	28,500	0.768 (0.757, 0.779)	0.520
	TRisk	Myocardial infarction	28,500	0.822 (0.812, 0.832)	0.583
Baseline medication	MAGGIC-EHR	Beta-blockers	34,258	0.751 (0.741, 0.761)	0.509
	MAGGIC-EHR+	Beta-blockers	34,258	0.753 (0.743, 0.762)	0.511
	TRisk	Beta-blockers	34,258	0.801 (0.792, 0.810)	0.564
	MAGGIC-EHR	Angiotensin-converting-enzyme inhibitors	52,799	0.769 (0.761, 0.777)	0.502
	MAGGIC-EHR+	Angiotensin-converting-enzyme inhibitors	52,799	0.769 (0.761, 0.777)	0.502
	TRisk	Angiotensin-converting-enzyme inhibitors	52,799	0.811 (0.804, 0.819)	0.553
	MAGGIC-EHR	Angiotensin receptor blockers	26,205	0.751 (0.740, 0.762)	0.544
	MAGGIC-EHR+	Angiotensin receptor blockers	26,205	0.752 (0.741, 0.763)	0.542
	TRisk	Angiotensin receptor blockers	26,205	0.805 (0.795, 0.815)	0.600

HF: heart failure; AUPRC: area under precision-recall curve; CI: confidence interval

Table S16. Multivariable risk models for patients with heart failure

Authors	Risk prediction model	Derivation cohort					Validation	
		Sample size	Country/ region	Patient population	Outcomes investigated	Variables included	# Patients; Setting	Metrics
Levy et al. (2006) [2]	Seattle Heart Failure Model	1,125	USA and Canada	Patients with symptomatic systolic HF (i.e., with reduced EF)	1-, 2-, and 3-year risk of all-cause mortality	Diuretic dose, SBP, lymphocyte, haemoglobin, uric acid, allopurinol use, LVEF, ischaemic aetiology, sodium, NYHA class	2,987; international [2]	AUC: 0.68 (0.65-0.71); Observed vs predicted (deciles) with correlation of 0.97
							148; USA [2]	AUC: 0.81 (0.72-0.90); Observed vs predicted (deciles) with correlation of 0.98
							925; USA and Canada [2]	AUC: 0.68 (0.63-0.73); Observed vs predicted (deciles) with correlation of 0.99
							5,010; international [2]	AUC: 0.69 (0.68-0.72); Observed vs predicted (deciles) with correlation of 0.97
							872; Italy [2]	AUC: 0.75 (0.70-0.80); Observed vs predicted (deciles) with correlation of 0.99
							10,930; USA[3]	AUC: 0.66; Hosmer-Lemeshow test: $\chi2 = 8.7$, P = 0.36
							6,161; international[4]	AUC: 0.714 (0.691-0.736); Observed-to-predicted ratios: 0.98; Hosmer-Lemeshow test: P < 0.001
Pocock et al. (2013) [5]	Meta-analysis Global Group in Chronic Heart Failure Model	39,372	International (30 cohorts)	Patients with diagnostic HF (i.e., both reduced and preserved EF)	1-, and 3-year risk of all-cause mortality	Age, EF, NYHA class, creatinine, diabetes, beta-blocker use, SBP, BMI, HF duration, current smoker, COPD, gender, ACE-I or ARB use	10,930; USA[3]	AUC: 0.69; Hosmer-Lemeshow test: $\chi2 = 38.6$, P < 0.001
							6,161; international[4]	AUC: 0.743 (0.720-0.766); Observed-to-predicted ratios: 1.03; Hosmer-Lemeshow test: P < 0.001
							39,372; internal [5]	Graphical discrimination and calibration of observed vs predicted (deciles); quantitative metrics NA
							6,263; international [6]	AUC: composite of cardiovascular death or HFH: 0.60 (0.58-0.63); cardiovascular death: 0.65 (0.61-0.69); all-cause mortality: 0.63 (0.60-0.66)
							5,625; Korea [7]	AUC: 0.73; Hosmer-Lemeshow test: P = 0.56
							51,043; Sweden [8]	AUC: 0.74; Observed-to-predicted ratios: 1.08
McDowell et al. (2024) [6]	Prognostic Models for Mortality and Morbidity in Heart Failure With Preserved Ejection Fraction Models	6,263	International	Patients with symptomatic HF (i.e., both reduced and preserved EF)	1-, and 2-year risk of cardiovascular death or HFH; cardiovascular death; all-cause mortality	NT-proBNP level, HFH within the past 6 months, creatinine level, diabetes, geographic region, HF duration, treatment with a sodium-glucose cotransporter 2 inhibitor, chronic obstructive pulmonary disease, transient ischemic attack/stroke, any previous HFH, and heart rate	4,796; international [6]	AUC: composite of cardiovascular death or HF: 0.71 (0.69-0.74); cardiovascular death: 0.70 (0.65-0.74); all-cause mortality: 0.67 (0.63-0.71)
							4,128; international [6]	AUC: composite of cardiovascular death or HF: 0.75 (0.73-0.78); cardiovascular death: 0.75 (0.71-0.79); all-cause mortality: 0.74 (0.70-0.77)

ACE-I: angiotensin-converting enzyme inhibitor; ARB: angiotensin-receptor blockers; AUC: area under the curve; BMI: body mass index; COPD: chronic obstructive pulmonary disease; EF: ejection fraction; HFH: heart failure hospitalization; HF: heart failure; LVEF: Left ventricular ejection fraction; NT-proBNP: N-terminal pro–brain natriuretic peptide; NYHA: New York Heart Association; ROC: receiver operating characteristic; SBP: systolic blood pressure; NA, not applicable;
